# Integrated coupled assessment of geostorage and geothermal prospects in the oil fields of Upper Assam Basin

**DOI:** 10.1038/s41598-024-60292-3

**Published:** 2024-05-29

**Authors:** Anupal Jyoti Dutta, Nababrot Gogoi, Firdush Zallah Hussain, Sandeep D. Kulkarni

**Affiliations:** 1 Deysarkar Centre of Excellence in Petroleum Engineering, Indian Institute of Technology, Kharagpur, West Bengal 721302 India; 2 Oil India Limited, Duliajan, Assam 786602 India; 3 IFP School, 228-232 Av. Napoléon Bonaparte, 92852 Rueil-Malmaison, France

**Keywords:** CO_2_ storage, Geothermal energy, Monte Carlo simulation, Geochemometrics, Upper Assam Basin, Solid Earth sciences, Energy science and technology

## Abstract

This study proposes an integrated approach of assessing CO_2_ storage potential and geothermal energy prospect based on the data of seventeen depleted wells of Upper Assam Basin which could assist the global objective of net zero transition. The petrophysical properties of Tipam, Barail and Lakadong + Therria Formations from the seventeen wells have been utilised to perform the Monte Carlo simulation for probabilistic estimation of the CO_2_ storage in the Upper Assam Basin. This preliminary work showed that the mean storage capacity of 18.8 ± 0.7 MT, 19.8 ± 0.9 MT and 4.5 ± 0.8 MT could potentially be stored in the three geological formations of the basin. The corrected bottom hole temperature values for the studied seventeen wells were determined using the well log data and Waples and Harrison method; these values provided a static geothermal gradient for each well, which varies widely from 0.017 to 0.033 °C/m. In order to enable geothermal prospectivity, static formation temperature maps have been generated for the studied wells. The probabilistic assessment of stored heat-in-place and formation temperature maps delimited five prospective sites for the extraction of geothermal energy in the basin. The study also presented a risk assessment for CO_2_ storage development in the basin. Further, the study illustrated an economic analysis of the implementation of a CO_2_ storage project and geothermal operations in the basin.

## Introduction

The global need to decarbonize the energy sector and facilitate a transition to renewable energy sources is evident. To combat climate change and reduce carbon emissions, it is crucial to implement more renewable energy solutions and adopt net-zero emission strategies across all sectors. Carbon dioxide (CO_2_) storage and geothermal technologies are currently in focus for facilitating the energy transition in India. The life cycle assessment (LCA) studies have demonstrated that, the diesel and steel consumption, required for the construction of any new geothermal wells, is the main factor responsible for related environmental impact^1^. Despite this environmental impact, the hot spot analysis performed over the life cycle of several enhanced geothermal power plants demonstrated that the carbon intensities of the geothermal energy production were atleast 50 times lower than the fossil-based technologies^2,3^. The LCA analysis of flash steam and dry steam based geothermal production also showed significantly lower carbon intensities than the fossil-based technologies except for the cases that involved unstable methane emission^4^.

In recent years, there has been a growing interest in geostorage and geothermal technologies to bring about a tectonic shift in the hydrocarbon industry^5–14^. These technologies offer innovative solutions for energy storage and the extraction of heat from the Earth, with the potential to transform the way we produce and consume hydrocarbon. Carbon capture and storage *(CCS)* technology emerges as a promising method to decrease CO_2_ emissions. Geological sequestration of carbon dioxide (CO_2_) is considered a viable solution for mitigating greenhouse gas emissions^15–17^. Storing CO_2_ in depleted hydrocarbon fields or reservoirs is considered an effective and economical option, with the potential to store or reinject a significant amount of CO_2_ and contribute to emission reduction targets^18–21^. Recent advancements in the assessment of CO_2_ storage in existing and depleted oil and gas reservoirs have been successfully conducted in various countries, including the US, Canada, Australia, China, the North Sea region, etc.^22–24^. India has huge potential, around 291 Gt^7^, to store CO_2_ across various sedimentary basins and could strengthen the global sustainable development goal to limit to 2 °C. However, in India, primary studies indicate substantial storage capacity in the sedimentary basins, including the Assam Shelf and Assam-Arakan fold belt^7^. However, there is a lack of studies on storage capacity estimation in the depleted fields of the Upper Assam Basin, located in Northeast of India. This study aims to assess the storage potential in the three sedimentary formations (Tipam, Barail, Lakadong + Therrria) of the Upper Assam Basin utilizing the petrophysical properties of the selected wells in the region.

**Figure 1:**
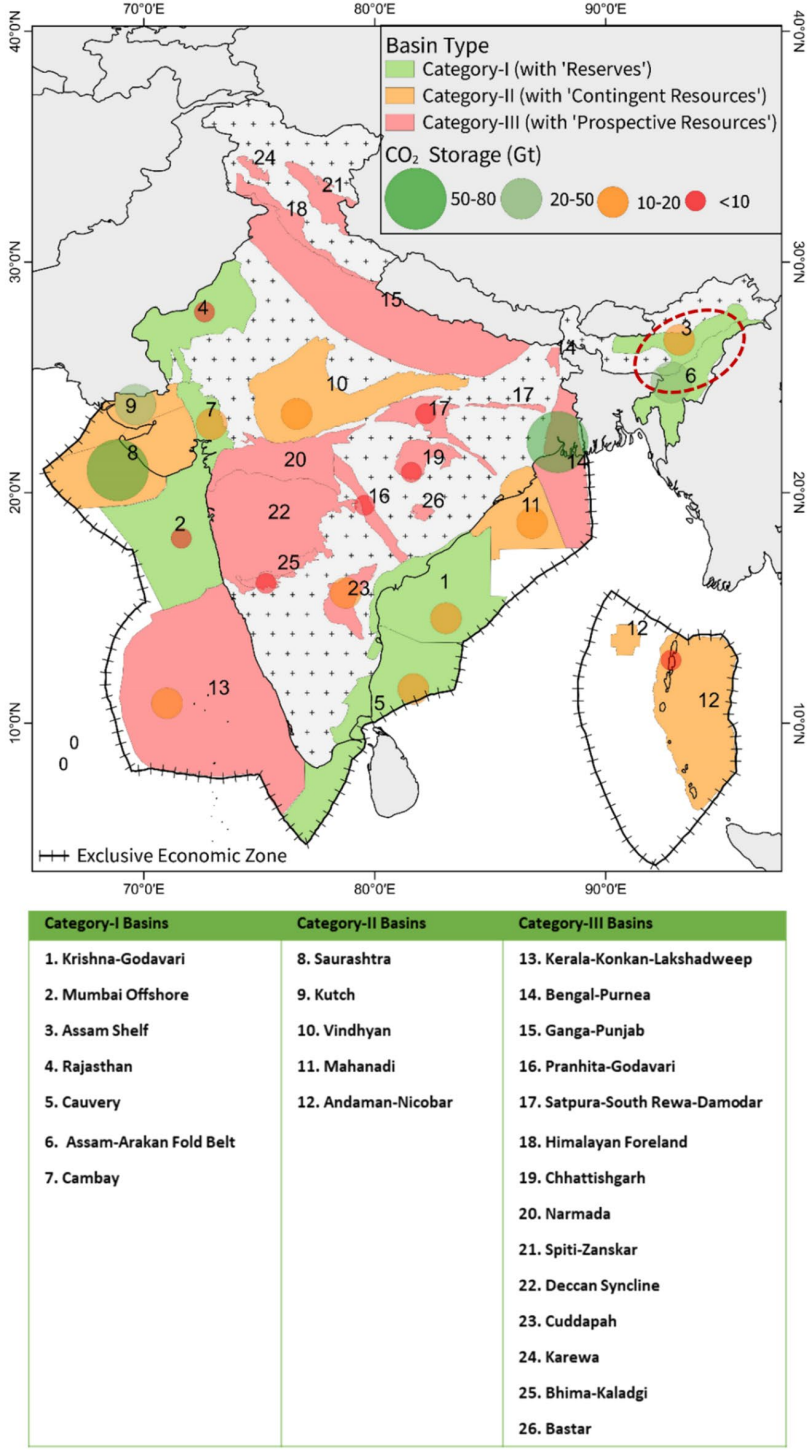
Indian sedimentary basinal map showing potential CO_2_ storage sites with the respective estimated storage amounts (tonnes) in circles – map designed based on earlier literature data^3^ (QGIS3.36 https://www.qgis.org/en/site/forusers/download.html).

**Figure 2:**
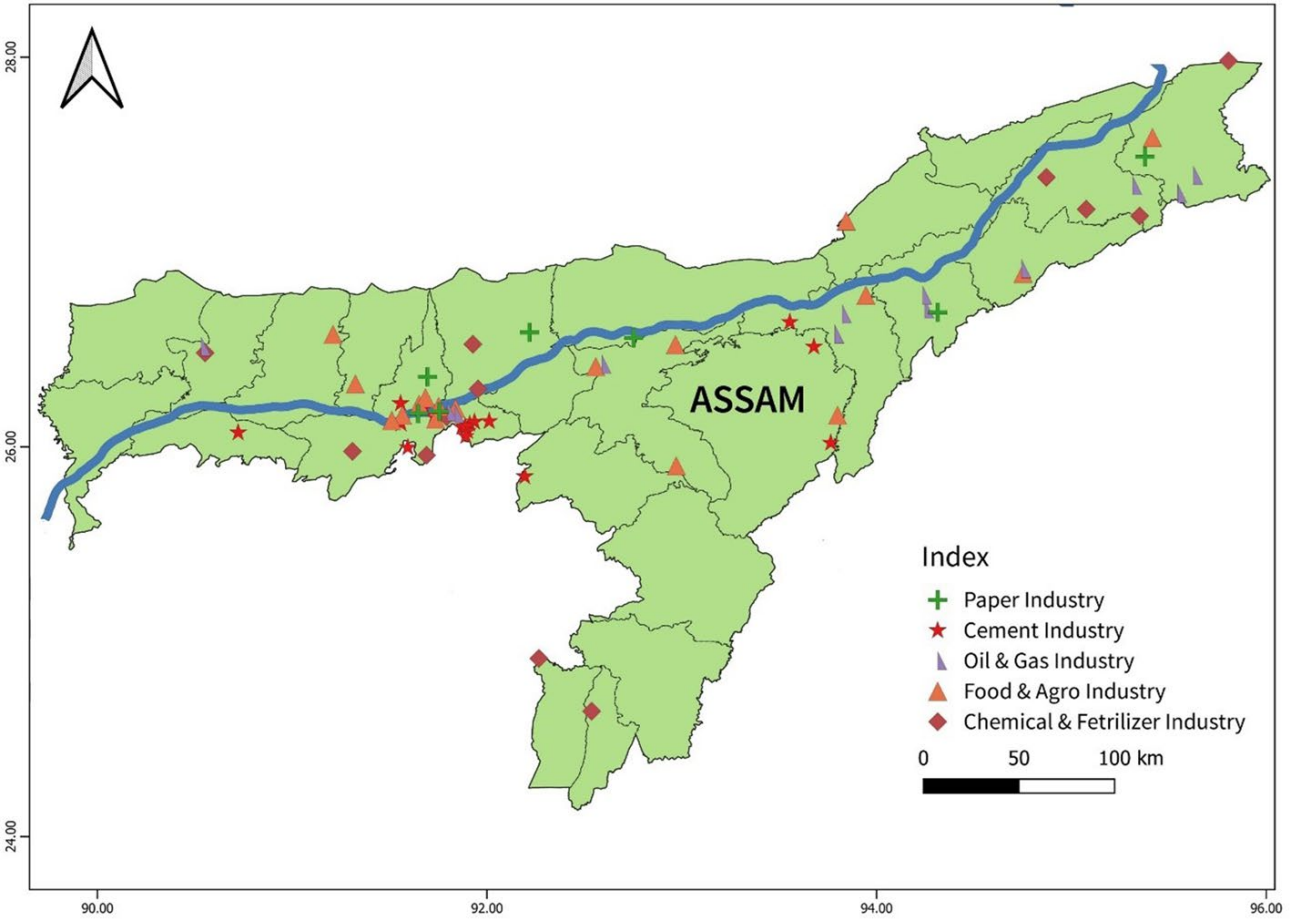
Map showing the numerous point sources of CO_2_ for the studied area in Assam. (QGIS3.36 https://www.qgis.org/en/site/forusers/download.html).

In the Indian subcontinent, there exist several potential storage basins where CO_2_ capture and storage (*CCS*) projects could be implemented. These basins, which may include the Category-I and Category-II basins of India^7,25^, differ in their geological characteristics and site suitability for long-term CO_2_ storage. Figure 1 illustrates the potential storage capacity across various sedimentary basins of India. Primary contributers of CO_2_ emissions in the Indian subcontinent include industrial facilities, power plants, transportation and other large-scale operations that utilize fossil fuels (coal 54%, oil 37% and gas 6%) leading to release of significant amounts of CO_2_ (≈ 2.65 Gt /per year)^26^ into the atmosphere. A few of these CO_2_ emission sources in the vicinity of the selected wells for the study area is shown in Fig. 2.

The objective of this study is to estimate the capacity of storing CO_2_ in oil fields of Assam-Arakan Basin. The Assam Shelf is geographically placed within significant CO_2_ point sources such as IOCL Digboi (production capacity ≈ 1 MMTPA), BVFCL Namrup (production capacity ≈ 600 MT/year of urea-II/ ammonia-II), BCPL Dibrugarh (production capacity ≈ 0.22 million TPA and 0.06 million TPA of polyethylene and polypropylene), thermal power plants, cement industries etc. shown in Fig. 2. The comprehensive assessment of CO_2_ storage capacity presented in the current study would assist in tapping these CO_2_ sources for storage in the depleted oilfields of the Upper Assam Basin. Accurate and well-documented calculations of CO_2_ storage resources are necessary for governments to assess the viability of storing CO_2_ in the sub-surface environment, and subsequently commercial organizations developing site specific CCS programs.

The Upper Assam Basin is categorised as a category-1 petroliferous basin in North-East India^27^. This study is aimed to assess both the CO_2_ storage potential in the three sedimentary formations (Tipam, Barail and Lakadong + Therria) and geothermal prospectivity in the Lakadong + Therria Formation of the basin. It exhibits a promising geothermal energy potential related to abandoned or depleted oil and gas fields, which are influenced by subsurface heat fluxes. The repurposing of abandoned oil and gas wells for geothermal energy sector is beneficial from the environmental standpoint as, with this approach, the construction of separate geothermal wells and associated carbon footprint can be avoided. Repurposing abandoned wells for geothermal energy is also cost effective as it would reduce the drilling cost and consequently the overall capital cost of the plant^5,10,14^. Retrofitting or repurposing of abandoned or high water cut-producing wells as geothermal wells are feasible due to the presence of attractive/favorable high bottom hole temperature (BHTs) at few of the depleted wells in the area^6^. Given that most oilfield reservoirs operate on water drive mechanisms, large volumes of hot water are produced without utility, posing environmental hazards. Exploration of these high-temperature fields along with the usage of produced water volumes for geothermal energy can play a significant role in achieving India's net-zero goal by 2070. This study aims to evaluate the thermal characteristics of different oil wells to effectively utilize potential geothermal heat stored or available in the region.

## Geological settings

**Figure 3:**
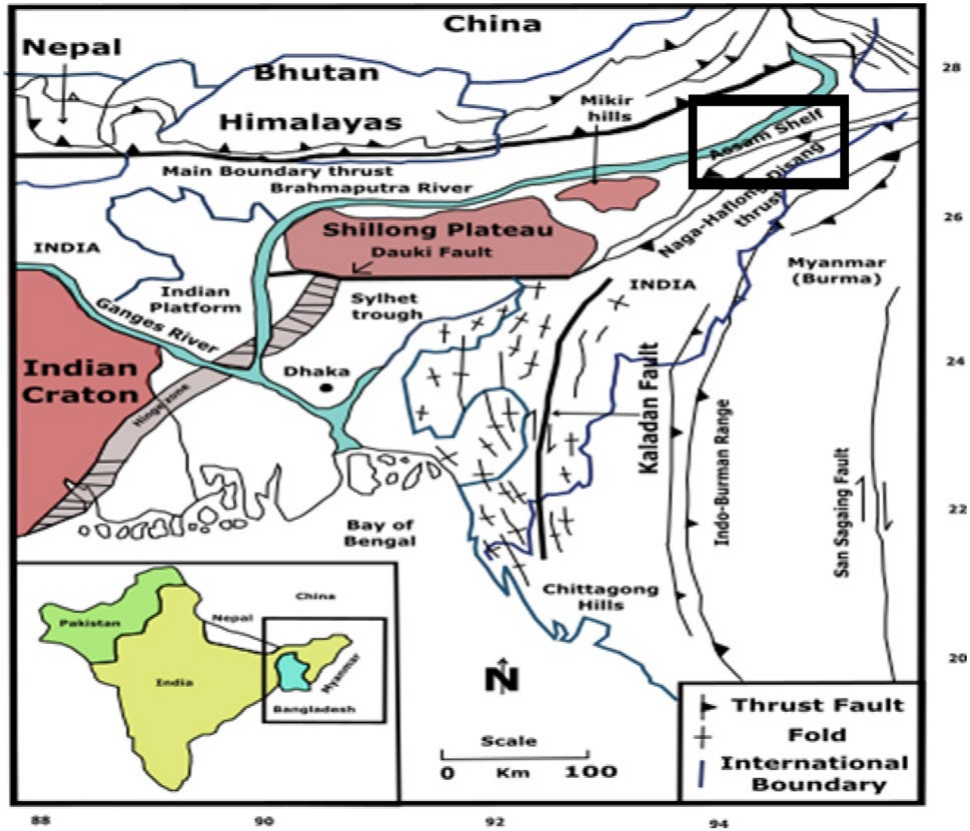
A tectonic map^30^ and the study area in the Upper Assam Shelf as highlighted in the box with black outline. (QGIS3.36 https://www.qgis.org/en/site/forusers/download.html).

The Upper Assam Basin primarily derives its oil and gas production from formations in the Upper Assam Shelf, which is bounded by three major thrust zones: the Himalayan orogenic thrust belt in the north, the Mishmi Thrust in the east, and the Schuppen (Naga-Disang thrust belt) Belt in the south (Fig. 3). The Assam Shelf foreland Basin, characterized by its topography, represents a normal floodplain area formed by the river Brahmaputra and its tributaries. However, the alluvial plains of Assam exhibit a wide arc-shaped formation at the basement level, aligning with the path of the Brahmaputra River.

The basin's geological history is complex, involving multiple phases of tectonic movements. It initially started as an extensional basin and later experienced compression phases associated with the Indo-Eurasian collision. The tectonic evolution of the basin is often described as an "oblique collision" and tectonic wedging model. Various researchers have contributed to the understanding of the stratigraphic disposition in the Upper Assam Basin, including Medlicott^28^, Mallet^29^, Evans^30,31^, L. L. Bhandari and R. C. Fuloria^32^, Rangarao^33^, and others. The Thanetian beds of the Therria Formation are often combined and referred to as the Lakadong + Therria Formation (Lk + Th). This grouping is primarily due to the absence of well-defined lithological and paleontological characteristics, as well as their relatively limited thickness^34^. The generalised Tertiary stratigraphic succession is shown in Table 1.

**Table 1:** Generalised stratigraphic sequence of Upper Assam Basin.

Age		Group	Formation	
Pleistocene		Alluvium		
Tertiary	Pliocene	Dihing Group	Dhekiajuli Formation	
	Miocene	Dupitila Group	Namsang Formation	
		Tipam Group	Girujan Formation	
			Tipam Formation	
	Eocene–Oligocene	Barail Group	Argillaceous Unit	
			Arenaceous Unit	
	Eocene	Jaintia Group	Kopili Formation	
			Sylhet Formation	Prang Member
				Nurpuh Member
				Lakadong + Therria (Lk + Th)
	Palaeocene	Langpar Formation		
Precambrian	Basal sandstone and Basement complex			

## Materials and methods

**Figure 4:**
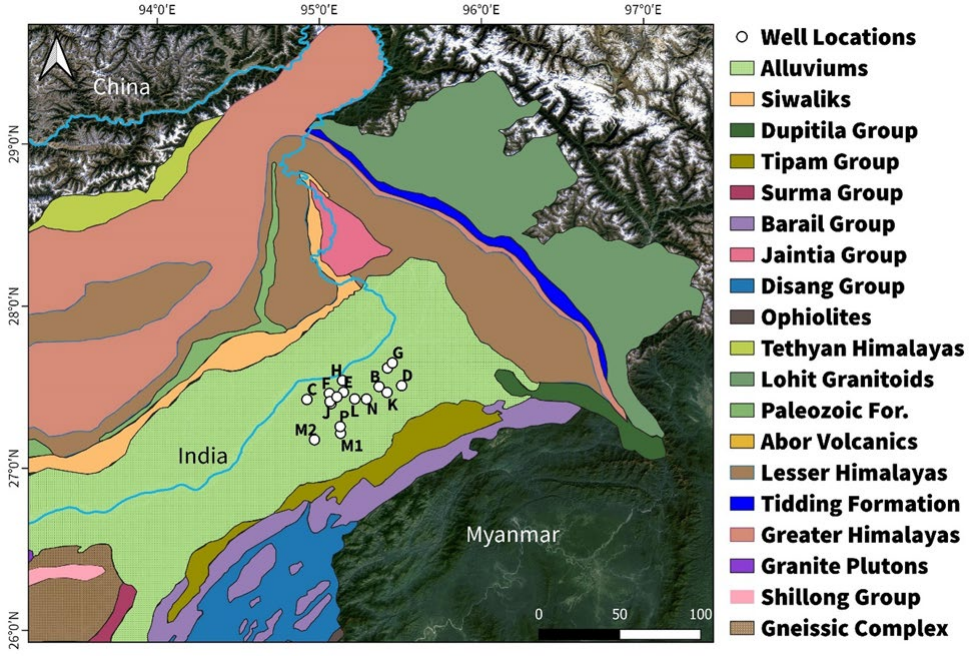
Geological Map indicating locations of selected wells of Upper Assam Basin modified after GSI (1998) & Long et al. (2011). (QGIS3.36 https://www.qgis.org/en/site/forusers/download.html).

A detailed petrophysical analysis was conducted on seventeen specific wells located within the operational areas of the Upper Assam Shelf as shown in Fig. 4. The focus was on evaluating the petrophysical properties of the studied formations, namely the Tipam, Barail and Lakadong + Therria Formations of the Upper Assam Basin. The petrophysical characterization was estimated using Techlog wellbore software (SLB) from the available well logs labelled as Gamma Ray *(GR*), Resistivity (*LLD*), Density *(RHOB)* and Neutron-Porosity *(NPHI)* log of the study area. The characterization involved identifying the porous and permeable zones, estimate porosity (*ϕ*) from porosity logs (NPHI, neutron and density tool), recognize hydrocarbon and a water bearing zones from resistivity logs, and then applying Archie’s relationship to find formation water resistivity (*R*_*w*_) and water saturation (*S*_*w*_), and the respective data table is presented in the supplementary section.

Previous research works have employed the Monte Carlo simulation approach^24,35^ to estimate the theoretical storage capacity^36,37^ of a saline aquifer.The analysed petrophysical data was considered as input parameters to perform a Monte Carlo simulation to develop a probabilistic model for estimating the CO_2_ storage capacity in the depleted oil fields or reservoirs. The simulation considered a triangular statistical distribution of the input parameters by considering the probable (*P10*), possible (*P50*), and inferred (*P90*) petrophysical characteristics to calculate the theoretical storage capacity.

By utilizing this approach, the study aimed to provide a more comprehensive understanding of the potential CO_2_ storage capacity in few of the selected formations of the Upper Assam oil fields under examination, considering the uncertainty associated with the petrophysical properties as presented in the following sections.

### Basin suitability

The storage formations of interest in the oil and gas fields have depths ranging from 1800 to 4603 m, which aligns with the ideal depth for CO_2_ storage, with a minimum requirement of 800 m^38^. At depths below 800 m, the natural temperature and fluid pressures exceed the critical point (T = 31.1 °C, P = 7.38 MPa) of CO_2_ for most locations on Earth^39^. This means that injected CO_2_ at this depth or deeper will remain in a supercritical state due to the prevailing temperatures and pressures.

Seismic sections in the study area reveal the presence of normal faults as described in the later part of this work. These faults are a result of the Indo-Eurasian collisional tectonics, which have contributed to the formation of traps in most of the oil and gas fields in the Upper Assam Basin. The geological faults may act as natural pathways for CO_2_ migration; however, since the basin hosted and trapped oil and gas for several thousand years, the depleted oil and gas wells can be considered for securely storing CO_2_ in the basin.

**Figure 5:**
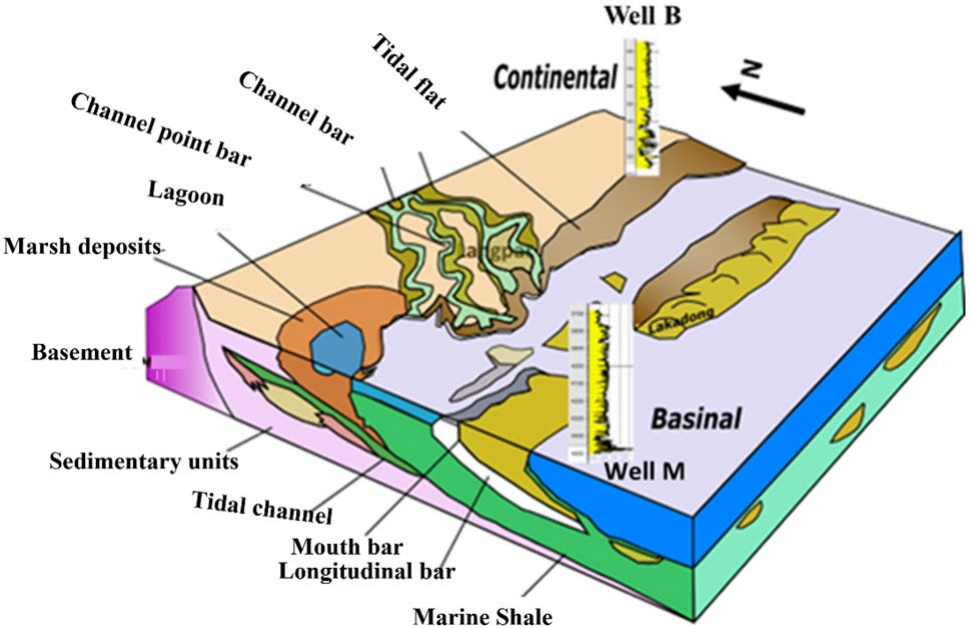
Modified Depositional facies^18^ of the Lakadong Eocene interval.

The geothermal conditions in the region exhibit a general increase in temperature with the rise of basement configuration. Earlier examination^40^ of the distribution of geothermal energy in the Upper Assam Basin revealed that anticlines and other regional geological formations are where the high concentration of energy may be found. These anticlines are frequently connected to deep-seated faults and basement highs (Handique and Bharali^40^). The availability of subsurface data, with the existing petroleum play in the basin, proves valuable in identifying prospective sites, including depleted and stranded fields in the Shelf as shown in Fig. 5.

### Site characterization

Previous studies conducted in the Upper Assam Shelf have suggested that CO_2_ enhanced oil recovery (*EOR*) techniques can serve as the initial step towards geological carbon storage, as it helps alleviate the financial burden associated with infrastructure development^7^. The objective of the current study is to evaluate the potential for CO_2_ storage in specific subsurface formations of interest.

**Figure 6:**
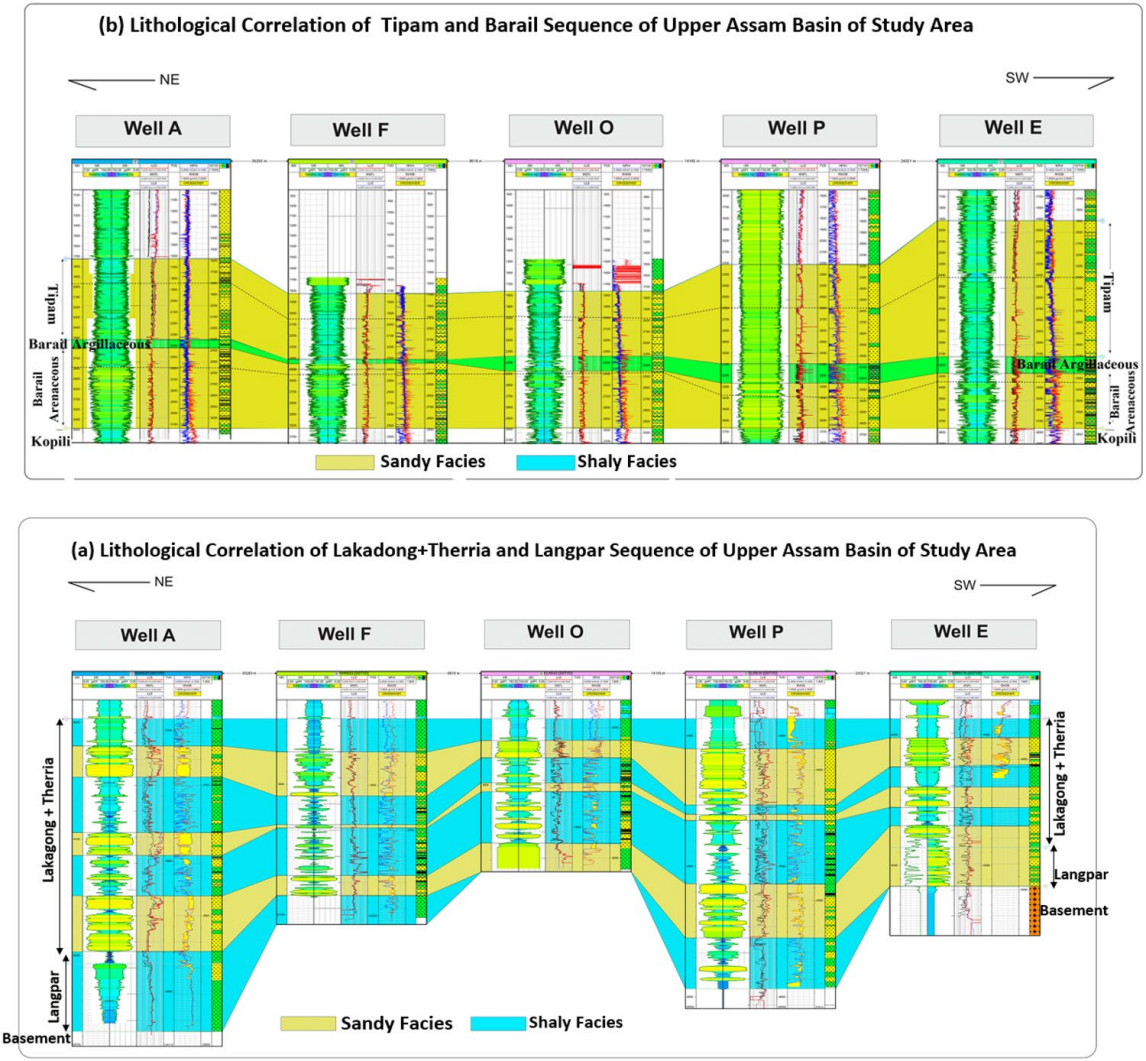
Lithofacies correlation of the selected wells across the Upper Assam shelf utilizing available Gamma Ray (GR), Resistivity (LLD), Density (RHOB) and Neutron-Porosity (NPHI) Log of the study area.

To facilitate this assessment, a lithofacies-based correlation for the studied formations (Fig. 6) has been established using available well-log data in the Upper Assam Basin. These formations exhibit lateral continuity along the Shelf, with thinning layers observed at the hinges where the basement rises. The petrophysical analysis have identified three major geological formations (Tipam, Barail and Lakadong + Therria) with projected CO_2_ storage potential in the Upper Assam Basin and their petrophysical characteristics are illustrated in Table 4.

The Tipam Formation is predominantly composed of sandstones with intermittent clay and shale layers (Bharali and Borgohain^41^). Its depositional environment during the Miocene was characterized by a braided river system, leading to the development of sheet sands (Bharali and Borgohain^41^). The Barail Formation exhibits two dominant facies: a Upper argillaceous facies and a Lower arenaceous facies. It was deposited in an Upper delta plain environment with fluvial influences^42^. The Lakadong + Therria Formation underwent lagoon-barrier island time transgressive sedimentation, with most of the oil reservoirs situated within the Barrier Island sands^43^.

**Figure 7:**
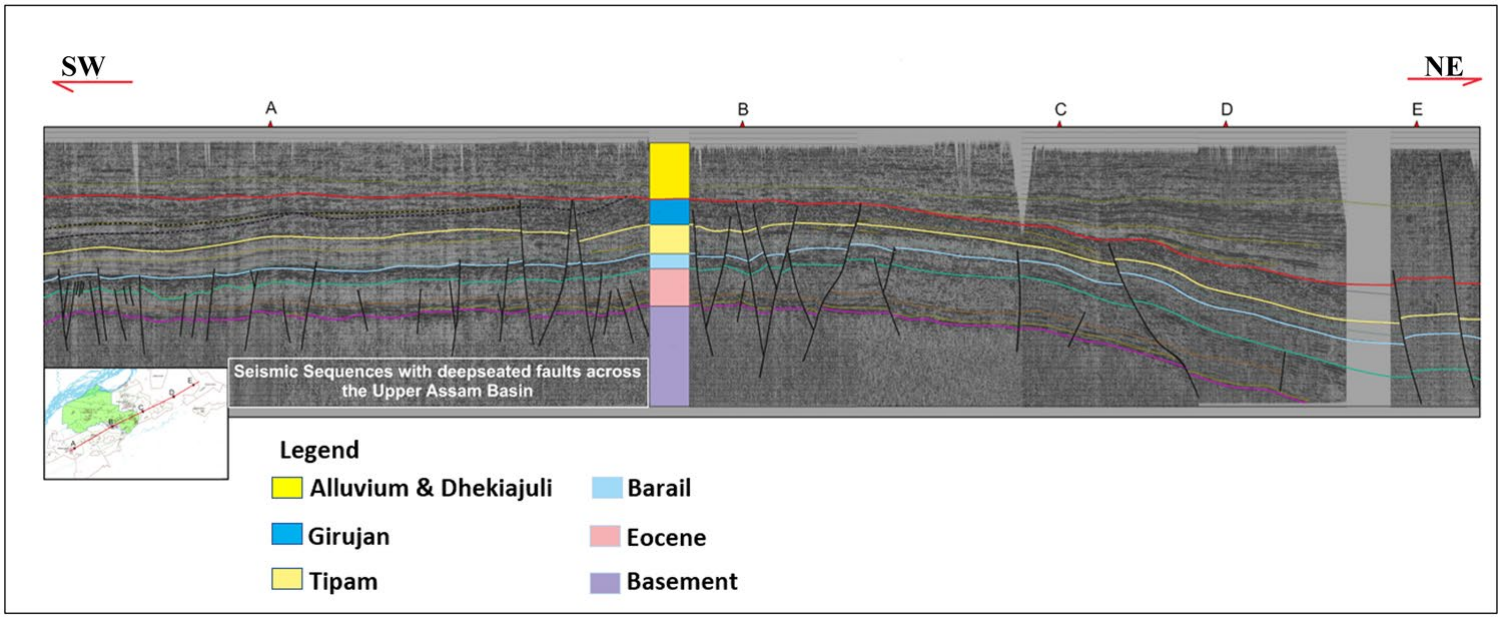
Regional structure and stratigraphy based on a NE-SW regional transect across the study site showing the Tertiary seismic sequence of five representative wells (A, B, C, D, E) in the Upper Assam Basin.

The analysis of 2D seismic lines in Petrel software (SLB) along the regional NE-SW transect indicates the uninterrupted nature of the Tipam and Barail Formations. Additionally, due to limited resolution, the Eocene reservoirs, including Lk + Th and Langpar Formation, are grouped together in the depicted section (Fig. 7). It is important to highlight the significance of basement faults and normal faults as observed in the seismic facies. The presence of these discontinuities and their potential impact on geothermal applications within the basin require further investigation, considering other crucial aspects such as wellbore integrity and reservoir heterogeneity.

### Assessment for CO_2_ storage capacity

The *CSLF* (Carbon Sequestration Leadership Forum) model closely resembles the model used by the US Department of Energy (*DOE*), with the main difference being the sequence of calculations. In the *CSLF* model^44^, the total theoretical storage capacity is determined first, followed by the application of a capacity coefficient between 0 and 1. The estimation of CO_2_ storage capacity in the formations involves calculating the net volume of suitable storage formations, and considering an appropriate storage efficiency factor. The efficiency factor considers various reservoir properties such as porosity, relative permeability, lithology, and other specific factors related with the in-situ pressure and temperature conditions. This approach helps in estimating the theoretical storage capacity of CO_2_ in the given formations^12^_._

The theoretical storage capacity (*SCTH*)^16,45^ is calculated as:

1 $$SCTH = GRV*\phi *NTG*\left( {1 - S_{wir} } \right)*\rho_{{CO_{2} }}$$

where *GRV* is gross rock volume, *S*_*wir*_ is irreducible water saturation, *ρ* is density of CO_2_ as a function of temperature and pressure (*T,P*) and *NTG* is the net to gross ratio of the formations. The *GRV*, *ϕ* and *NTG* for the studied seventeen wells were obtained based on the well-log data and available in the supplementary section. The effective or usable CO_2_ storage capacity^45^ is given by:

2 $$SCO_{2} = \, SCTH*E$$

where *E* is the storage efficiency factor that ranges between 0 and 1. The ‘*E*’ value depends on various formation parameters including *NTG* (net-to-gross), area, thickness, effective porosity, volumetric displacement (*EV*) as well as the microscopic displacement efficiency. Table 2 provides the values of ‘*E*’ used in the current study, based on the work of Goodman et al^37^; this literature study had estimated the efficiency factors for various lithologies (i.e. clastics, dolomite and limestone) in US and Canadian basins which were further conformed for other regions by various researchers^46–48^. The efficiency factors with two significant figures were as reported in Goodman et al.^37^ for the clastics, dolomite, and limestone lithologies using log-odds normal distribution.

**Table 2:** Saline formation efficiency factors (reference) (E) used for the current study.

Lithology	P10 (%)	P50 (%)	P90 (%)
Clastics	0.51	2.0	5.4
Dolomite	0.64	2.2	5.5
Limestone	0.40	1.5	4.1

### Estimation of S_wir_ irreducible water saturation

Several empirical methods^49–51^ have been established to correlate the porosity (*Φ*), permeability *(K*) of the formation with irreducible water saturation (*S*_*wir*_); one such generalized correlation (Eq. 3) was particularly chosen for this study as it was validated in the literature^52^ using well log data in the various dominant lithologies of concern (shale, sandstone, limestone) in the studied area. Equation 3 estimates the irreducible water saturation (*S*_*wir*_) for the formations as:

3 $$S_{wir} = \frac{C}{{\frac{\phi }{{\left( {1 - V_{cl} } \right)}}}}$$

**Figure 8:**
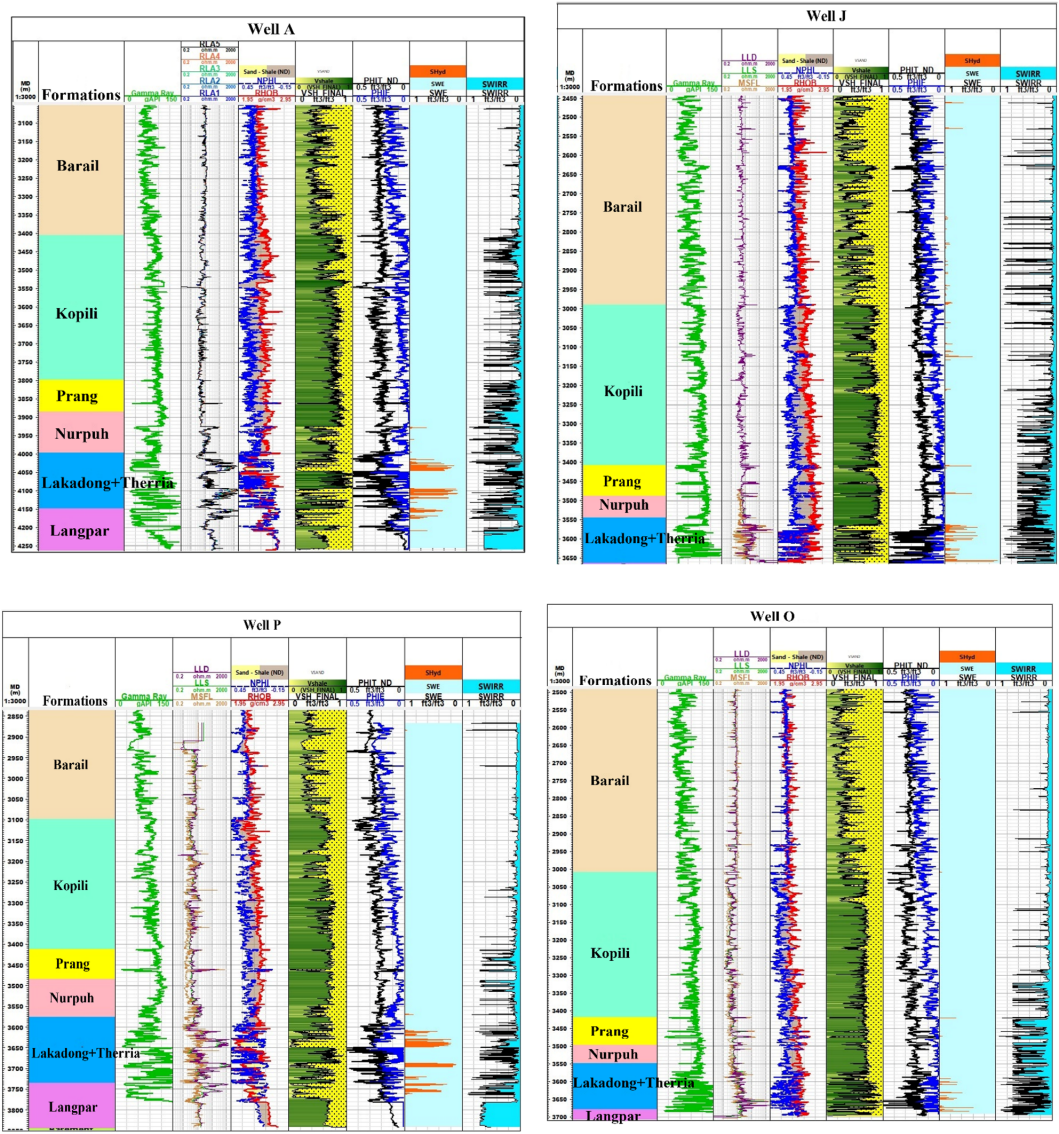
Petrophysical analysis of four representative wells (A, J, P, O) utilizing available Gamma Ray Log (GR), Resistivity Log and Neutron-Density (NPHI) Log of the study area.

*C* is Buckle’s constant and *V*_*cl*_ is the volume of clay. By employing Eq. (3), *S*_*wir*_ for the Tipam, Barail and Lk + Th Formation were determined by using petrophysical properties obtained from the well logs of the seventeen wells. Figure 8 indicate the estimation of* S*_*wir*_ of four representative wells on the basis of the estimated petrophysical properties using Techlog wellbore software (SLB). The* S*_*wir*_ calculated for Tipam Formation showed value of 0.07 ± 0.65 (µ ± σ); while for the Barail Formation the *S*_*wir*_ variation was 0.18 ± 0.75 and in case of the Lakadong + Therria Formation the *S*_*wir*_ variation was estimated as 0.19 ± 0.64.

### Geothermal regime

The discussed seventeen oil and gas wells, drilled in the Upper Assam Basin, recorded bottomhole temperatures ranging from 60 to 120 °C for depths varying from 3579 to 4603 m (Table 3). Subsequent works^6,40^ on the subsurface geothermal maps demonstrated a consistent trend of increasing temperatures with the upliftment of the basement configuration. The higher temperatures are observed over the crests of local highs, gradually decreasing towards the flanks. The geothermal gradients tend to be relatively higher in arenaceous sediments compared to argillaceous sediments, as indicated by the earlier study^40^. In the literature, the Upper Assam Basin was shown to exhibit an average heat flow value of 61 mW/m^2^ ^53^. This heat flow value indicates a low-to-medium enthalpy field similar to the Assam Shelf, as noted by Razdan et al.^54^.

**Table 3:** Calculation of geothermal gradient for the selected seventeen wells.

Selected Wells	Raw BHT (°C) @ Well depth (m)	Corrected BHT (°C) (Harrison Method)	Corrected BHT °C (Waples Method)	Corrected Geothermal gradient (°C/m)—G_t_
A	95.0 @ 4045	114	115.3	0.022
B	85.0 @ 3978	104	102.7	0.019
C	100.6 @ 3952	119.6	122.7	0.024
D	97.6 @ 4420	116	117.5	0.021
E	93.0 @ 3708	112	113.8	0.023
F	73.8 @ 3758	92.8	89	0.017
G	75.0 @ 4300	93.6	89.1	0.015
H	85.0 @ 3994	104	102.7	0.019
I	94.7@ 3652	113.6	116.2	0.025
J	116.0 @ 3579	134.8	143.8	0.033
K	102.0 @ 4165	120.8	123.8	0.023
L	111.7 @ 3613	130.5	138.2	0.031
M1	115.0 @ 4547	133	139.2	0.025
M2	113.0 @ 4283	131.7	137.5	0.026
N	90.6 @ 4031	109.6	109.7	0.021
O	94.0 @ 3915	113	114.4	0.026
P	114.0 @ 4603	131	137.7	0.024

**Figure 9:**
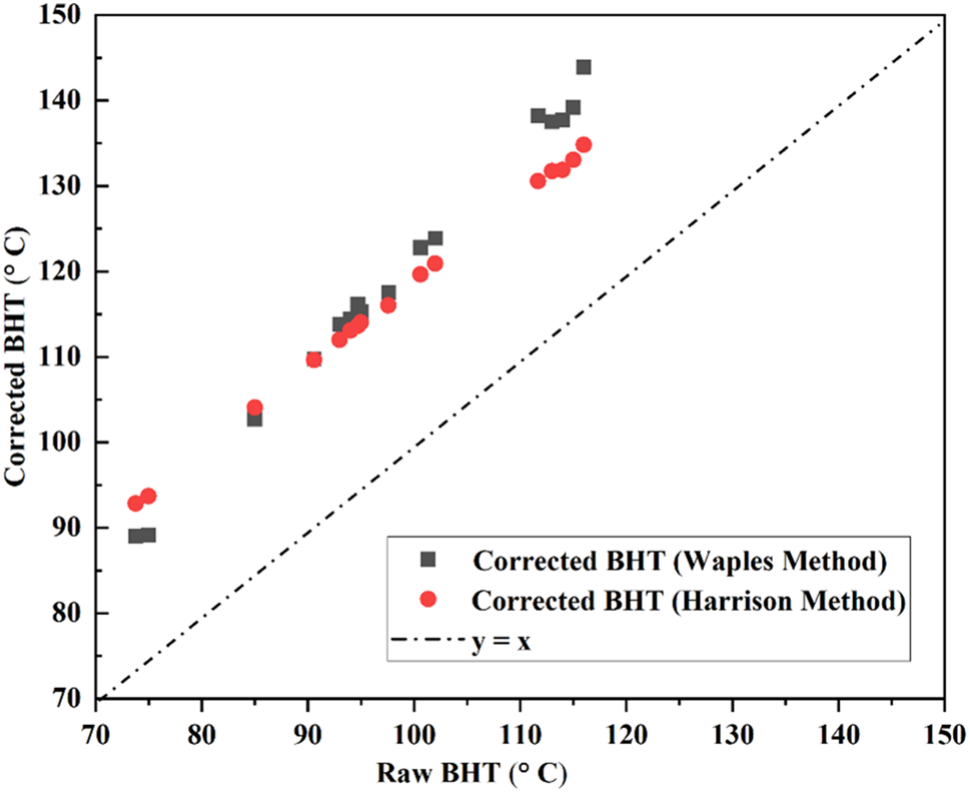
BHTs of the selected seventeen wells in the study area corrected after Harrison^59^ and Waples^60^ Method.

It was noted that the actual undisturbed/equilibrium BHTs of the reservoir may vary from the recorded well-log BHTs depending on the reservoir characteristics and the wellbore operational parameters. The factors such as temperature of drilling fluid temperature, time and pumping rate, shut-in time, borehole radii, and thermal diffusivity of the borehole need to be considered to develop such correlations and model the true static formation temperature (SFT)^55–58^. Owing to the limited data availability from this basin, the raw well-log BHTs have been corrected in this study to obtain the far-field formation temperatures in the Upper Assam oil fields using the Harrison method^59^ and Waples method^60^ as shown in Table 3; the same has been plotted as corrected vs. uncorrected in Fig. 9.

#### Geothermal gradient

**Figure 10:**
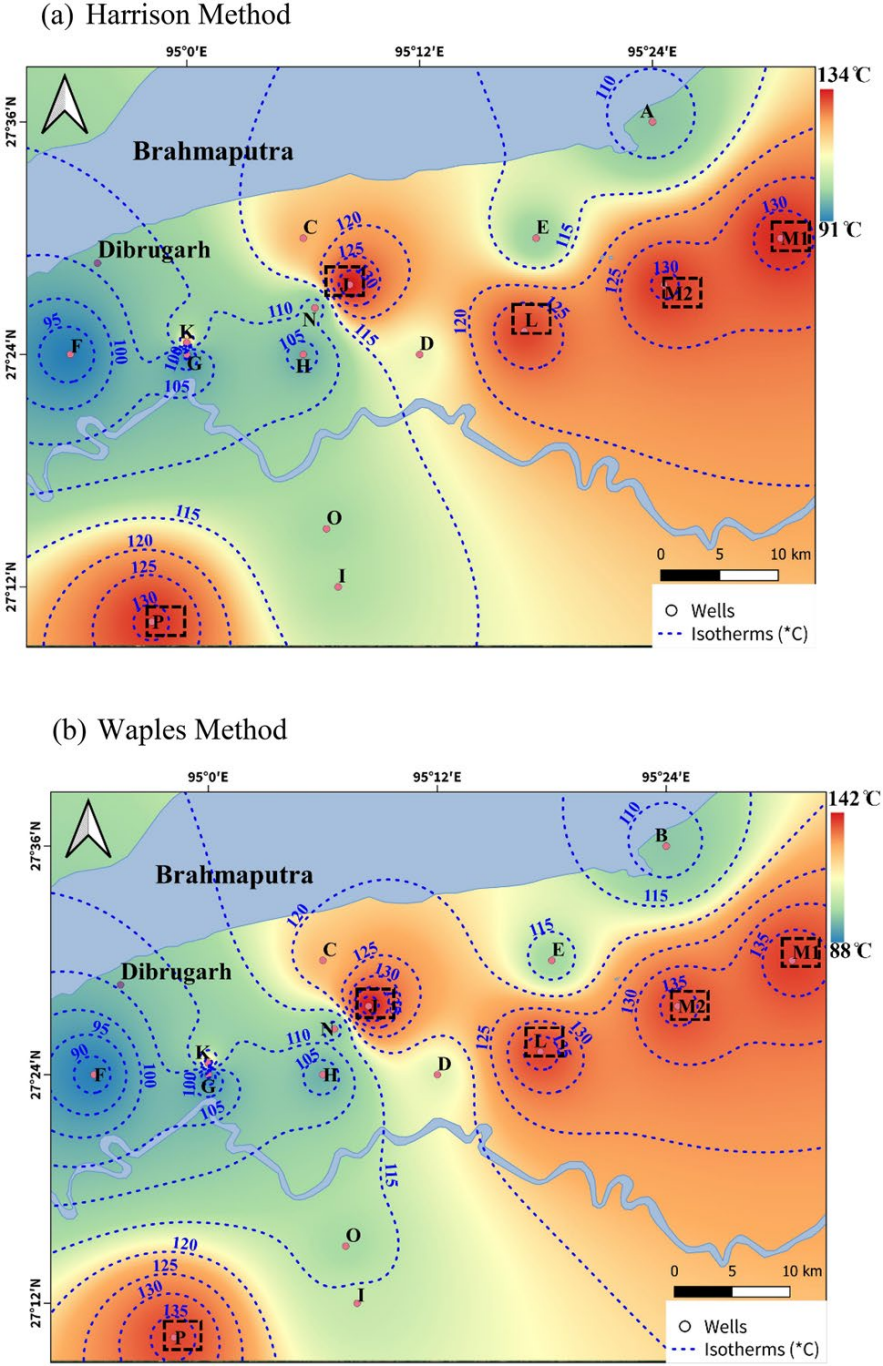
Iso-Temperature Map ((**a**) Harrison Method (**b**) Waples Method) of the selected wells of the Upper Assam Basin with five prospective well sites of Lk + Th Formation for geothermal plants). (QGIS3.36 https://www.qgis.org/en/site/forusers/download.html).

The geothermal gradients in the studied area were determined by applying a simplified linear equation as:

4 $${\text{T}}_{{\text{f}}} = {\text{ T}}_{{\text{s}}} + {\text{ G}}_{{\text{t}}} *{\text{ Z}}$$

where *T*_*f*_ is the formation temperature at the corresponding depth of the formation (*Z*), *T*_*s*_ is the surface reference temperature (24 °C) and geothermal gradient is represented by *G*_*t*_. Using the corrected BHT data (Harrison method^59^ and Waples method^60^) and well depth information of the specified seventeen wells, the corrected geothermal gradient was determined as shown in Table 3. The reported uncertainty in the corrected BHT values was 6–8%^58^; the same has been incorporated in the assessment of heat-in-place evaluation in the later section. The derived values of geothermal gradient were utilized to determine the formation top temperature maps of the Lk + Th Formation using software *QGIS 3.36* shown in Fig. 10a,b and accordingly five prospective well sites have been identified as potential wells for pilot scale field studies to assess the geothermal potential of this particular formation in the Upper Assam Basin.

(*Z* = measured depth (m); BHT = Bottom Hole Temperature (°C)).

*BHT Correction Method

$$\begin{aligned} & \left. {\begin{array}{*{20}l} {\Delta T = - 16.51213476 + 0.01826842109*Z - 2.344936959*10^{ - } 6*Z^{2} } \hfill \\ {{\text{T}}_{{\text{c}}} = \, \Delta {\text{T }} + {\text{ T}}_{{\text{m}}} } \hfill \\ \end{array} } \right\}{\text{Harrison}}\;{\text{Method}} \\ & \left. {\begin{array}{*{20}l} {{\text{T}}_{{\text{c}}} = {\text{ T}}_{{\text{s}}} + {\text{ f}}*\left( {{\text{T}}_{{\text{m}}} {-}{\text{ T}}_{{\text{s}}} } \right)} \hfill \\ {{\text{f }} = \, \left( { - 0.{1462}*{\text{ln}}*\left( {{\text{TSC}}} \right) \, + { 1}.{699}} \right) \, / \, \left( {0.{572}*{\text{Z}}^{{0.0{75}}} } \right)} \hfill \\ \end{array} } \right\}{\text{Waples}}\;{\text{Method}} \\ \end{aligned}$$

where *∆T, f* is the correction factor, *TSC* is time since circulation in hrs, *Z* is measured depth (m).

#### Assessment of heat-in-place

The calculation of the heat-in-place takes into account various parameters of the reservoir including the specific heat capacity (*c*_*r*_, J/g °C), density (*ρ*_*r,*_ kg/m^3^), volume (*V*, m^3^), and temperature (*T*_*r*_, °C). The average temperature on the earth's surface (*T*_*s*_) is typically assumed to be around 24 °C for this calculation. The volumetric heat-in-place or dynamic stock, *S*_*o*_ is determined using the following equation:

5 $$S_{0} = \, \rho_{r} \cdot \, c_{r} \cdot \, V \cdot \, \left( {T_{r} - \, T_{s} } \right)$$

The above equation, proposed by and Hackstein and Madlener^61^, considers the density (*ρ*_*r*_) and heat capacity (*c*_*r*_) of the reservoir, along with the volume (*V*) and the difference in temperature (*T*_*r*_*—T*_*s*_). To account for the heat capacity of the reservoir and its porosity, the following equation proposed by Gringarten^62^ is used:

6 $$\rho_{r} \cdot c_{r} = \Phi \cdot \rho_{f} \cdot \, c_{f} + \left( {1 - \Phi } \right) \cdot \, \rho_{r} \cdot \, c_{r}$$

This equation, proposed by Gringarten and Sauty^63^ and Hackstein and Madlener^61^ incorporates the porosity (*ɸ*) of the reservoir, as well as the densities (*ρ*_*f*_*, ρ*_*r*_) and heat capacities (*c*_*f*_*, c*_*r*_) of the fluid and rock, respectively.

## Results and discussions

**Figure 11:**
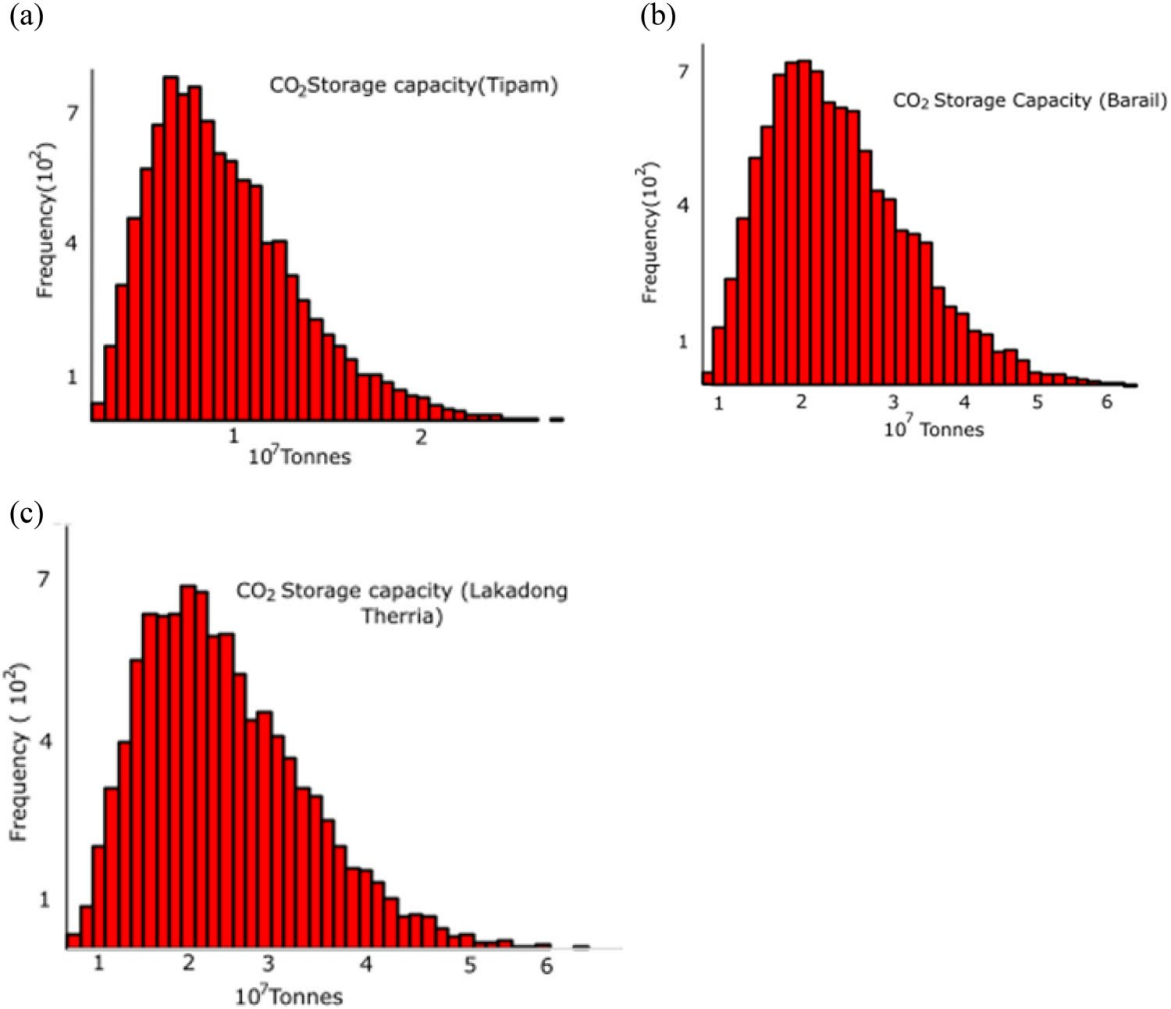
CO_2_ storage capacity using a Monte Carlo simulation for (**a**) Tipam prospect, (**b**) Barail prospect and (**c**) Lakadong + Therria prospect.

In the current study, a probabilistic model is developed, utilizing the Monte Carlo simulation technique. This simulation involves utilizing Eq. (1) with the input of the petrophysical properties (triangular distribution is assumed for the input properties in the wake of limited data) of the studied formations presented in Table 4 to perform 10,000 iterations of the Monte Carlo algorithm. The simulation performed with higher iterations (> 10,000) provided insignificant change in the outcome; for instance, the variation in the output was with ± 1% in case of 15,000 iterations. The simulations resulted in probabilistic output generation for the storage capacities of the studied formations as demonstrated in Fig. 11. The mean storage capacities (µ ± σ) for the three formations, namely Tipam, Barail and Lakadong + Therria in the studied area, are estimated to be 18.8 ± 0.7 MT, 19.8 ± 0.9 MT and 4.5 ± 0.8 MT respectively (MT—Million Tonnes); additionally, the storage uncertainties in terms of P10, P50 and P90 values are also illustrated in the Table 5.

**Table 4:** Petrophysical Properties of the studied formations.

Formation	Thickness (m)			NTG			ɸ			1-S_wir_			Area (km^2^)		
	P10	P50	P90	P10	P50	P90	P10	P50	P90	P10	P50	P90	P10	P50	P90
Tipam	500	300	200	0.9	0.8	0.7	0.45	0.30	0.20	0.81	0.75	0.65	12	13.5	7.6
Barail	600	500	400	0.85	0.75	0.70	0.35	0.22	0.18	0.82	0.75	0.70	12	13.5	7.6
Lakadong + Therria	160	120	90	0.9	0.8	0.7	0.30	0.20	0.16	0.8	0.7	0.6	12	13.5	7.6

**Table 5:** Theoretical CO_2_ storage capacity in tonnes against frequency using a Monte Carlo simulation for (a) Tipam prospect (b) Barail prospect and (c) Lakadong + Therria prospect for the studied wells in Upper Assam Basin.

Formation	Storage Capacity (MT-Million Tonnes)		
	Mean	Minimum	Maximum
Tipam	18.8	2.3	66.0
Barail	19.8	3.0	61.3
Lakadong + Therria	4.5	0.6	13.0

**Figure 12:**
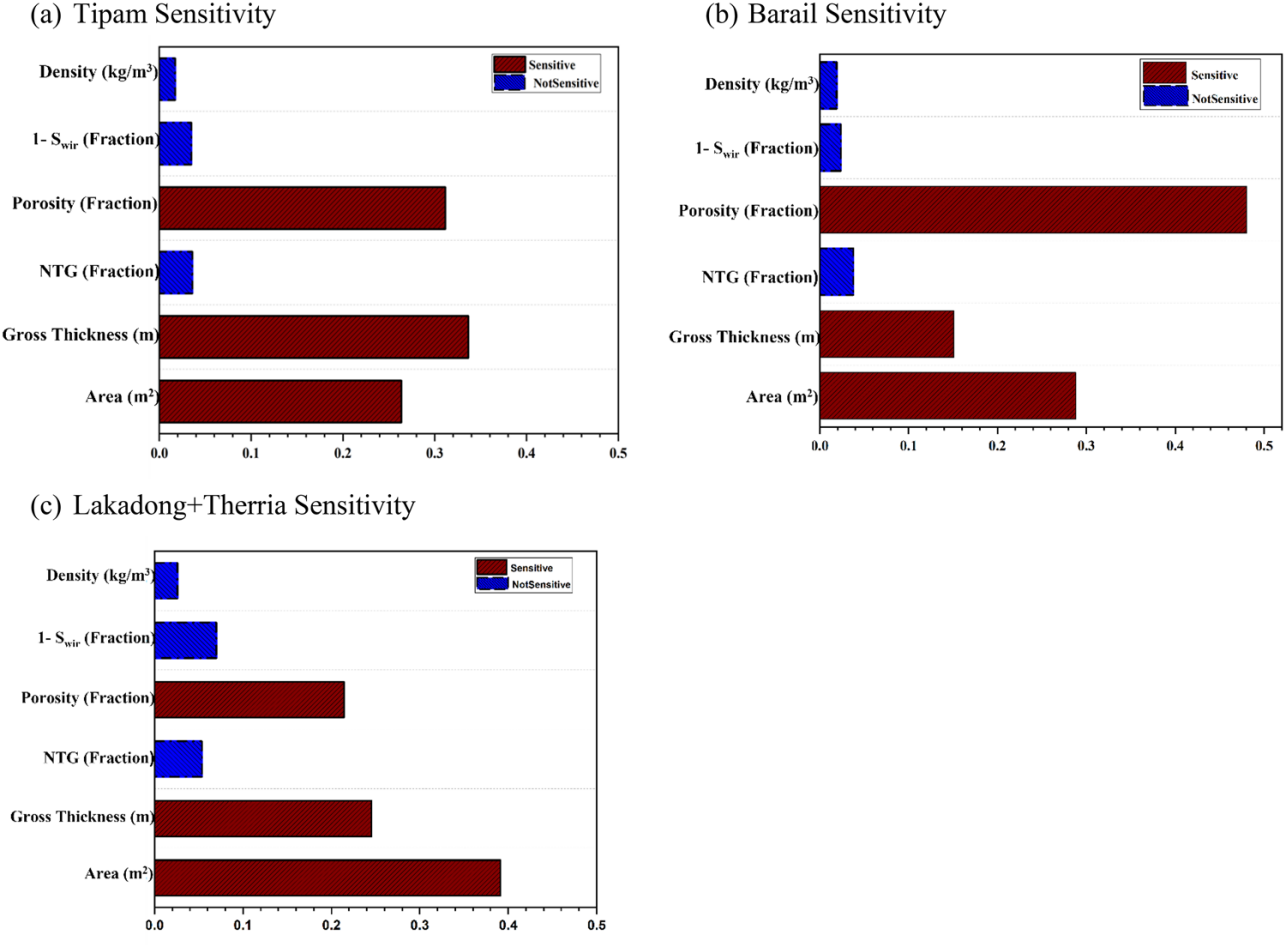
Sensitivity analysis of the input parameters for Monte-Carlo simulation for CO_2_ storage capacity estimation for (**a**) Tipam, (**b**) Barail & (**c**) Lakadong + Therria Formations in the Upper Assam Basin.

A relative impact plot shown in Fig. 12 is constructed for the three studied formations viz. Tipam, Barail and Lk + Th to know the contribution of the uncertainity of the individual input parameters towards the total uncertainity. From the above sensitivity analysis for the three formations, it is observed that the reservoir parameters like porosity, gross-thickness and area contributed most to the total uncertainity in the present Monte-Carlo simulation study^24,35^. The higher contribution of these parameters towards the total uncertainty stems from significant difference in their {P10, P90} values; for instance, the {P10, P90} porosity values of the Tipam formation {0.2, 0.45} indicate more than 100% variation. To the contrary, the uncertainty contribution of the NTG parameter is significantly less (Fig. 12) as its {P10, P90} values only show 30% variation for the studied formations as depicted in Table 4.

To assess the geothermal potential of the Upper Assam Basin, the information on Lk + Th Formation recorded bottom hole temperatures of the seventeen studied wells shown in in the Upper Assam Basin were utilized. The evaluation of the bottom hole temperature data of the studied wells in earlier section depicted that certain wells (M1, M2, L, J and P) provided higher geothermal gradient (> 0.024 °C/m); these wells were selected for the heat-in-place analysis as described below. The geothermal heat-in-place (*H. I. P*) at these five well sites for Lk + Th Formation were evaluated using Eqs. (5) and (6). The below Table 6 presents the calculated heat-in-place (*H. I. P*) within the reservoir, considering probabilistic areas at radial distances of 5 km (P10), 3 km (P50), and 1.5 km (P90) around the proposed sites.

**Table 6:** Geothermal Heat-in-Place assessment of the studied wells.

Selected wells	H.I.P (× 10^14^ J)		
	P10	P50	P90
M1	11.2	3.0	0.47
M2	11.1	2.9	0.47
L	13	3.6	0.6
J	10.8	3.1	0.45
P	11	2.9	0.46

**Figure 13:**
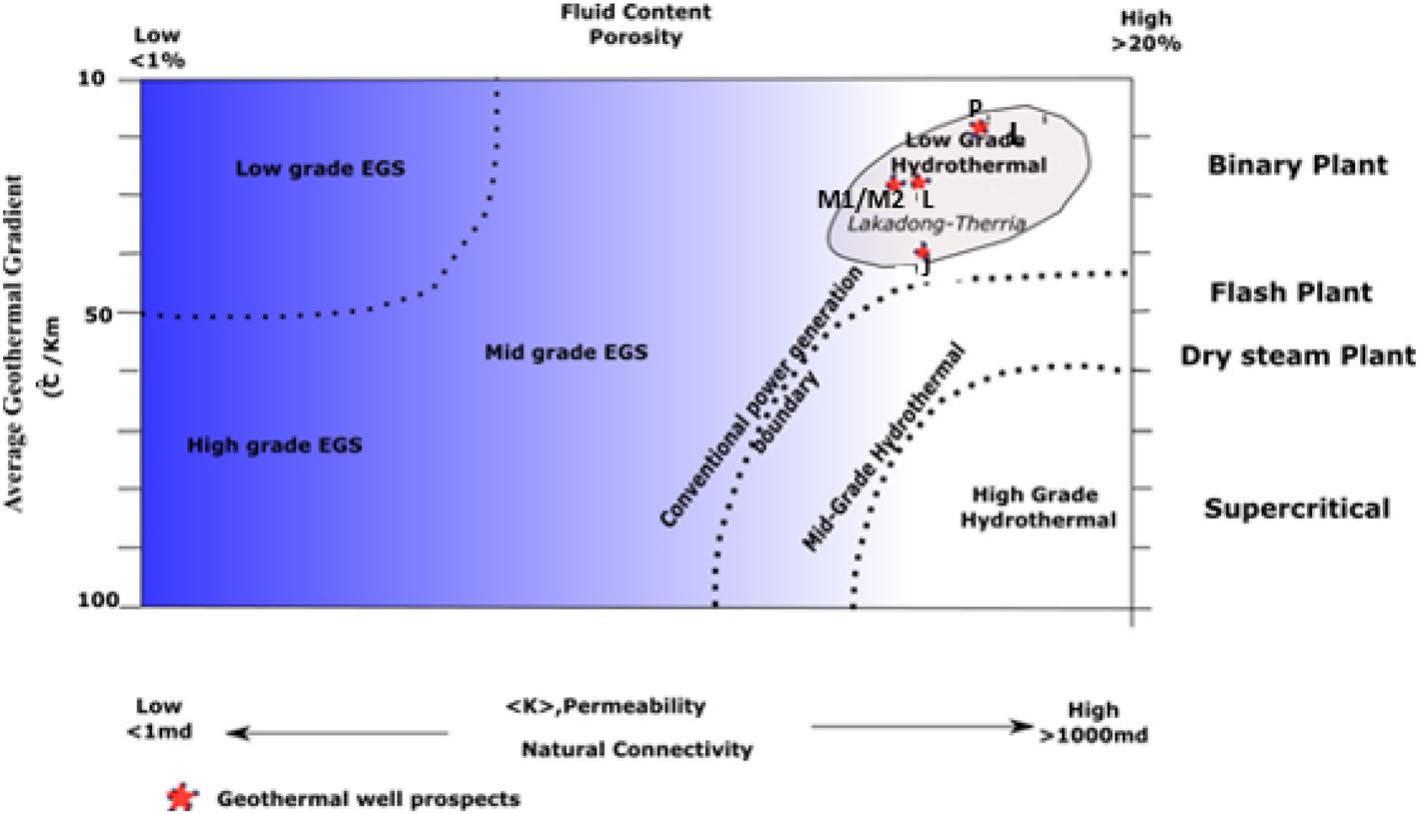
Geothermal heat extraction strategy plot for five prospective sites of Lk + Th Formation of Upper Assam Basin^65^_._

The results revealed that the five identified sites in the Lk + Th Formation exhibited cumulative geothermal potential of P50 (*H.I.P)* ≈15.5*10^14^ J. It was noted that these formations also possess significant heterogeneity^34,64^. To device strategies for extracting heat from these identified sites, the following parameters are of key importance: porosity, permeability and geothermal gradient^65^_,_ accordingly, the geothermal heat extraction strategy for studied five sites may be recommended based on the binary plant as depicted in Fig. 13.

### Risks assessment study

**Figure 14:**
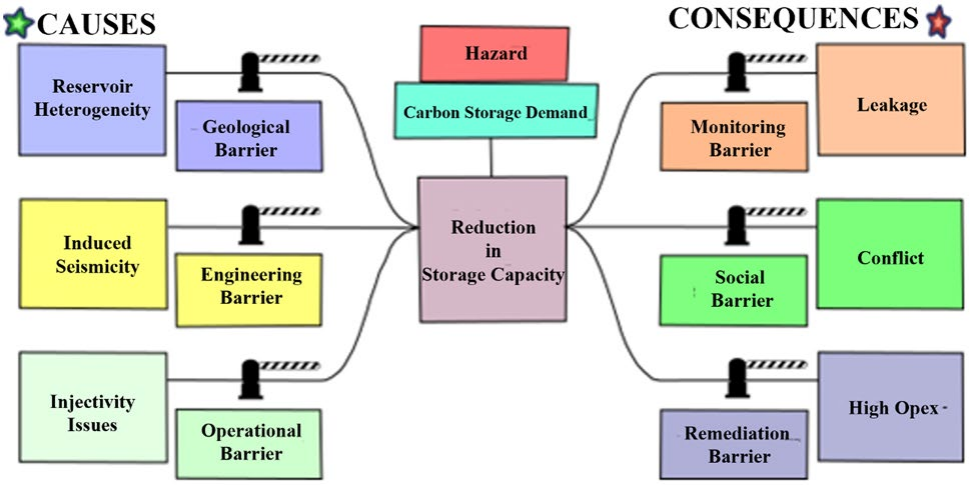
Risk Analysis for CO_2_ storage in selected formations of Upper Assam Basin^43^.

In order to effectively assess the risks associated with carbon capture, utilization, and storage (*CCUS*) in oil and gas fields in Upper Assam Basin, a thorough risk assessment is crucial. In this study, a rudimentary bow tie risk assessment is conducted to provide a qualitative evaluation of the hazards involved in this method. The bow tie diagram in Fig. 14, based on the work of Risktec Solutions Limited^66^ and Tucker et al.^67^, visually depicted the relationships between the origins of unwanted events, their potential outcomes, the preventive controls in place, and the mitigation mechanisms employed.

The starting point of the bow tie diagram is the "hazard", which refers to something within or around the organization that has the potential to cause damage. In this case, the identified hazard is an increase in demand for CO_2_ storage, as a decrease in storage capacity can significantly impact *CCUS* operations for subsurface CO_2_ sequestration. The next step is to define the "top event," which represents the moment when control over the hazard is lost, although damage or negative impacts have not yet occurred. In this study, the top event is identified as a reduction in CO_2_ storage capacity.

The left side of the top event comprises the causes or threats and their preventive barriers, while the right side represents the consequences and barriers to control them. Threats are the reasons that lead to the top event, and multiple threats can contribute to its occurrence. In this study for the Upper Assam Basin, three causes leading to the top event are identified:

- Reservoir Heterogeneity: The Upper Assam Basin reservoirs exhibit variability in petrophysical parameters as illustrated in Table 4 and can be affected by diagenetic perturbations, permeability baffles, and structural discontinuities like faults shown in the seismic section (Fig. 7). Detailed petrophysical characterization of the subsurface formation is necessary to assess heterogeneity and potential pathways for plume migration. Therefore, a geological barrier in the form of detailed subsurface characterization is needed to estimate heterogeneity and potential plume migration pathways. Geochemical reactions monitoring, which is critical to monitor the CO_2_ leaks, involves the tracing of CO_2_ at the surface or dissolved in groundwater. The geochemical sampling techniques could involve monitoring of the chemical variations, p^H^, water chemistry, etc. in produced groundwater^68^.
- Induced Seismicity: Induced seismicity, resulting from subsurface stimulation of hydrocarbon reservoirs, poses a common threat. In a seismically and tectonically active zone like Assam (Fig. 3), which is classified as seismic Zone-5, induced seismicity demands specific monitoring and extensive study before implementing any *CCS* projects. Along with induced seismicity, the quantification of geomechanical regime of the subsurface is crucial to identify potential earthquake-prone areas. Earlier studies^69^ indicate fault zonation should be performed to assess the stress regime and potential leakage pathways for CO_2_ plume migration during subsurface storage. Geophysical monitoring techniques need to be implemented at CO_2_ storage sites to monitor the leakage of CO_2_ through fractures, faults, structural discontinuities, etc. include the 2D, 3D seismic methods to detect the plume movement and migration pathway of CO_2_ in geological formations. Electromagnetic, electric, gravimetric, well logs are the other geophysical methods particularly useful in the monitoring of CO_2_ migration in geological formations^13,18^. Proper engineering barriers are necessary to prevent injectivity issues.
- Injectivity Baffles: Injectivity baffles^69,70^ can arise due to factors other than reservoir heterogeneity, such as compromised geopressure conditions and the geomechanical state of the subsurface. Regular inspection of the pipeline infrastructure is also necessary to prevent mineral precipitation and pipeline damage, which can reduce injectivity. Proper operational barriers for wellbore integrity in geological storage projects should be monitored to issues related to injectivity and leakage.

The consequences of the top event can be categorized based on project objectives. Some of the consequences discussed in this study include leakage, conflict, and higher operation expenses (*OPEX*) discussed in the later section. Continuous monitoring can help prevent leakage by detecting and addressing any damage. Socio-political conflicts resulting from such failures can be mitigated through proper socio-economic barriers, such as conducting social campaigns to maintain transparency between socio-political bodies. Higher operation expenses are expected in the event of a leakage, so remediation strategies should be established beforehand to enable prompt and effective action.

In summary, this rudimentary bow tie risk assessment highlights the potential risks associated with CO_2_ storage in oil and gas fields of Upper Assam Basin. By identifying the hazards, top events, causes, consequences, and barriers, it provides a qualitative understanding of the risks involved and emphasizes the importance of implementing preventive and mitigation measures to ensure safe and effective *CCUS* operations.

### Economics of CCUS in Upper Assam Basin

*CAPEX* (Capital Expenditure): The capital investment^71^ associated with any geostorage project mainly incorporates site exploration & site development, CO_2_ injection & monitoring, and abandonment. In the case of oil and gas fields, the site exploration phase is minimized as extensive study for reservoir parameters are investigated and available in literature. Site development involves converting existing wells into injection wells, and the *CAPEX* is negligible for injection. The estimated *CAPEX* for CO_2_ storage in depleted fields of Upper Assam is around $1.5–2 per tonne of CO_2_.

**Figure 15:**
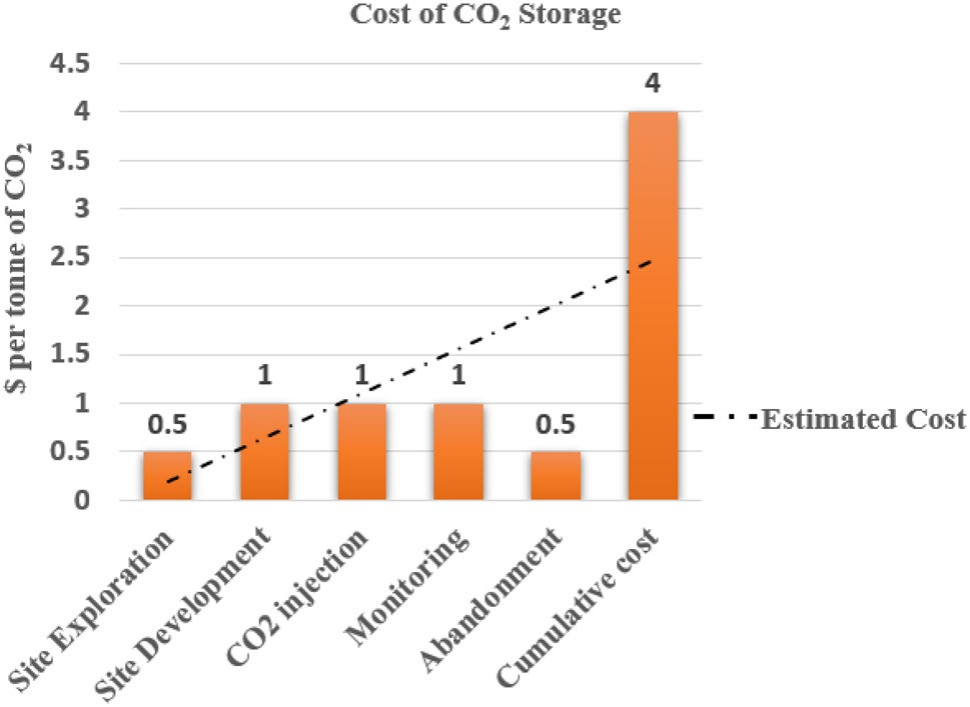
Estimated Cost^52^ for a CO_2_ Storage Site in Upper Assam Basin.

**Figure 16:**
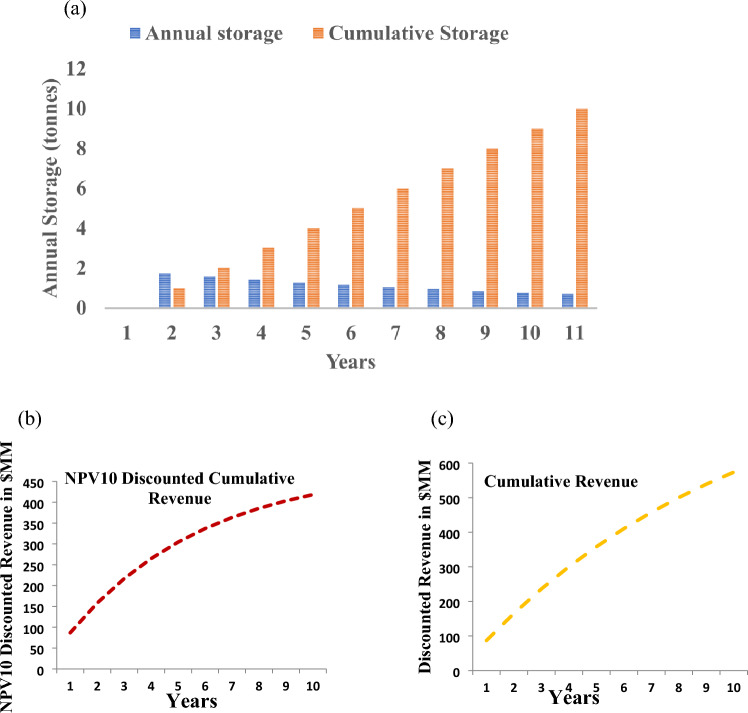
Total Cost model for Upper Assam oil and Gas fields (**a**) Storage Capacity (**b**) NPV Discounted Cumulative Revenue (**c**) Cumulative Revenue.

*OPEX* (Operating Expenditure): Operating costs^71^ include the monitoring and injection of CO_2_ in the subsurface. Based on the economic model^71^, the estimated *OPEX* is around $2–3 per tonne of CO_2_. The total cost incurred for CO_2_ injection is approximately $4 per tonne of CO_2_. Increasing costs linearly can further affect the time value of investments demonstrated in Fig. 15 below:

Site selection for CO_*2*_ geological storage requires site characterization work, which can be reduced when there is an existing oil and gas industry in the region. In the Upper Assam Basin, most of the storage prospects are within stranded and depleted oil and gas fields of the Naga Schuppen zone. The cumulative storage capacity, *NPV* Discounted Revenue, Cumulative Revenue for the selected wells of Upper Assam Basin have been estimated based on the earlier work^46^ and is shown in Fig. 16a–c.

The onshore saline aquifers in the Upper Assam Basin mainly consist of Neogene-Palaeogene sequences, including the Sylhet formation, Barail and Tipam. The CO_2_ for injection is sourced from point sources located throughout the basin. The current economic model does not include the cost of CO_2_ capture, which can vary depending on the specific *CCUS* policy and government regulations.

The mean storage capacity of the three formations within the depleted fields of the study area is estimated around 40 million tonnes. Assuming a mean storage capacity of 40 million tonnes and an optimum CO_2_ injection rate of 1.6 million tonnes per year over 10 years, the following costs were estimated. Net Present Value (*NPV*) was calculated to account for the depreciating value of investments. A discount rate of 5% is assumed over a 10-year period. However, at an optimum rate of 2 MT per year for a period of 10 years, as a base case scenario a storage project can approximately generate an *NPV* discounted revenue of 400 MM$ and cumulative revenue of 600 MM$ as shown in Fig. 16b,c.

It can be inferred from Fig. 16b, the *NPV* at the end of 10 years of injection is significantly lower than the substantial capital investments made during the project, indicating that the implementation of geological CO_2_ storage is currently uneconomical. To increase the commercial deployment of such projects, strong support from external funding agencies and government subsidies in the form of carbon tax credits is necessary to achieve the net-zero goal of India. Funding mechanisms such as carbon tax credits, which are currently $50 per tonne in the USA, can generate a net revenue with *NPV* of $1.4 billion. Direct capital grants can also be used to subsidize the *OPEX* incurred during injection. Initiatives like exemption from cess and royalty (as proposed in the Draft 2030 Roadmap for CCUS, 2022) can be starting steps to motivate implementation of pilot scale CO_2_ storage projects for industrial sector and further assist India achieving 2070 net zero target.

### Economics of geothermal energy

A typical geothermal power project incurs two types of costs: capital expenditure (*CAPEX*) and operating expenditure (*OPEX*). These costs can be further divided into surface and subsurface investments. Geothermal power generation can be achieved through three established technologies: dry steam, flash, and binary plants. Based on the temperature profile of the proposed sites, the appropriate technology in this case is the binary cycle. In binary geothermal power plants, a working fluid is employed in a closed cycle that is distinct from the geothermal fluid. The energy from the geothermal fluid is transferred to the working fluid through a heat exchanger, which then undergoes evaporation, expansion in a turbine, and condensation. The condensed fluid is pumped back to the heat exchanger. Binary plants commonly utilize Rankine or Kalina cycles^72^.

Studies by Chamorro et al.^73^ and Hackstein and Madlener^61^ have shown installation costs (*CAPEX*) ranging from $1000 to $3000 per kilowatt (*kW*) for a binary plant with an installed capacity of 1–35 megawatts (*MWe*). Hackstein and Madlener^61^ also provided an Eq. (7) to estimate per well costs (*C*_*well*_) based on the measured depth (*MD*) of the well.

7 $$C_{well} = (1.72 \times 10^{ - 7} \cdot \left( {MD} \right)^{2} + 2.3 \times 10^{{{-}3}} \cdot MD{-}0.62)0.10^{6}$$

where *C*_*well*_ (*US$*) are the well costs and *MD* (m) is the measured depth. To minimize costs, existing production wells can be repurposed as geothermal wells in the proposed sites, potentially eliminating the need for additional well costs. However, operation and maintenance costs (*OPEX*) are directly proportional to the energy produced and follow an exponential decline with increasing plant capacity^74^. Another Eq. (8)^73^ is utilized a to estimate specific operating costs (*CAPEX*) as a function of the installed capacity (W) of the plant as below:

8 $$C_{OPEX} = \, 20 \cdot exp \, \left[ { - 0.0025 \cdot \left( {W \, - \, 5} \right)} \right]$$

Thus, the operational expenditures (*OPEX*) exhibit an exponential decrease from 20 *US$/MWh* for a 5 *MWe* plant to 12 *US$/MWh* for a 200 *MW*_*e*_ plant^61^.

The revenue generated is determined by the amount of electrical output generated. Assuming an electricity price of approximately INR 8 per kilowatt-hour (*kWhr*) in India, the net undiscounted revenue (*R*) can be calculated^61^ as the product of the electricity price (e) and the electrical output (*E*_*el*_). For a base case scenario with 10% efficiency, an estimated ~ 4.2 *TWhr* heat-in-place has the potential to generate as high as ~ 33.6 billion INR of undiscounted revenue.

## Conclusions

By examining the potential of both geostorage and geothermal energy, the study aims to provide valuable insights into sustainable strategies for reducing the carbon footprint of oilfields in the Upper Assam Basin of India. The findings from this study can significantly contribute and initiate the energy transition pathways, enabling a tectonic shift from fossil fuel resources towards cleaner and more environmentally friendly energy resources in the region.

CO_2_ storage has the potential application in the depleted oilfields of the Upper Assam Basin, where conventional oil extraction methods are currently in practise. The study examines the CO_2_ storage potential of three formations (Tipam, Barail and Lakadong + Therria) by considering their lithofacies correlations and petrophysical properties. Using a probabilistic model that incorporates a Monte Carlo simulation, the study presents a triangular distribution that represents the storage capacities of three formations. The mean storage capacities of these formations are reported as 18.8 ± 0.7 MT, 19.8 ± 0.9 MT, and 4.5 ± 0.8 MT respectively. From the sensitivity analysis performed, the net thickness, porosity and area contributed about 95% to the total uncertainity. A rudimentary bow tie risk assessment has highlighted the potential risks associated with CO_2_ storage projects in oil and gas fields in the basin. By identifying the hazards, top events, causes, consequences, and barriers, it can provide a qualitative understanding of the risks involved and emphasized the importance of implementing preventive and mitigation measures to ensure safe and effective *CCS* operations. The economic analysis undertaken in this study has estimated the *CAPEX* for CO_2_ storage in the depleted oil fields of Upper Assam Basin to be around 1.5–2$/tonne of CO_2_ and the *OPEX* to be around 4$ per tonne of CO_2_.

The geothermal potential of the Upper Assam Basin is evaluated using well-log data from seventeen wells. The recorded bottom hole temperatures (*BHTs*) of the Lk + Th Formation have been corrected with (6–8)% uncertainity and further utilized to generate the formation temperature maps and calculated the mean (*H.I.P)* ≈15.5*10^14^ J. The formation temperature maps, have revealed localized areas of high geothermal heat downhole along the basement ridge. The distribution of heat is mainly influenced by the sub-surface's structural configuration. Based on these findings, five well sites are identified as having high significant heat flux in terms of stored *H.I.P*. These sites have the potential for geothermal applications which could serve as a basis for further exploration of geothermal hotspots for pilot scale studies for the production of geothermal energy in the basin. The economic analysis carried out has revealed a significant decrease in operating expenses (*OPEX*) from 20 *US$/MWh* for a 5 *MWe* plant to 12 *US$/MWh* for a 200 *MWe* plant. Furthermore, in a base case scenario with a 10% efficiency rate, it is estimated that the presence of approximately 4.2 *TWhr* of heat in the basin could generate approximately 33.6 billion INR of undiscounted revenue.

### Supplementary Information

Supplementary Information.

## Data Availability

The petrophysical analysis undertaken on the basis of well log data was tabulated in Supplementary section. The well log data that support the petrophysical analysis of this study is available from Oil & Gas Industry (*Oil India Limited, Duliajan, Assam, India*) and upon request to Mr. Nababrot Gogoi (nabagogoi@oilindia.in) who is one of authors of this paper.

## Abbreviations

CCS: Carbon Capture and Storage
CCUS: Carbon, Capture, Utilization and Storage
Lk + Th: Lakadong + Therria
EOR: Enhanced Oil recovery
CSLF: Carbon Sequestration Leadership Forum
DOE: Department of Energy
SCTH: Theoretical Storage Capacity
SCO_2_: Usable Storage capacity
E: Storage Efficiency Factor
GRV: Gross-Rock-Volume
NTG: Net-to-Gross
Φ: Porosity
ρ_r_: Density of Rock (kg/m^3^)
ρ_f_: Density of Fluid (kg/m^3^)
c_r_: Specific Heat Capacity of Rock (J/g °C)
c_f_: Specific Heat Capacity of Fluid (J/g °C)
K: Permeability (md)
S_wir_: Irreducible Water Saturation
W: Installed Capacity (MW_e_)
E_el_: Electrical Energy (MWh)
KWh: Kilowatt-hours
TPA: Tonnes Per Annum
MMTPA: Million Metric Tonness Per Annum
MW: Megawatt
MW_e_: Megawatt electric
NPV: Net present value
CAPEX: Capital Expenditure
OPEX: Operating Expenditure
ORC: Organic Rankine Cycle
C_well_: Costs per well
MD: Measured Depth
TWhr: Terrawatt hour
BHT: Bottom Hole Temperature
H.I.P: Heat In Place
NPV: Net Present Value
$: Dollar
MM$: Mega Million Dollar
INR: Indian Rupees

## References

[1] Tomasini-Montenegro C, Santoyo-Castelazo E, Gujba H, Romero RJ, Santoyo E (2017). Life cycle assessment of geothermal power generation technologies: An updated review. Appl. Therm. Eng..

[2] Karlsdottir MR, Heinonen J, Palsson H, Palsson OP (2020). Life cycle assessment of a geothermal combined heat and power plant based on high temperature utilization. Geothermics.

[3] Kürten, S., Feinendegen, M., Noel, Y.F., Gaschnitz, R., Schwerdt, P., Klein, A. Geothermal utilization of smouldering mining dumps as a substitute for fossil fuels.

[4] Li J, Tarpani RRZ, Stamford L, Gallego-Schmid A (2023). Life cycle sustainability assessment and circularity of geothermal power plants. Sustain. Prod. Consum..

[5] Noorollahi Y, Pourarshad M, Jalilinasrabady S, Yousefi H (2015). Numerical simulation of power production from abandoned oil wells in Ahwaz oil field in southern Iran. Geothermics.

[6] Majumdar D, Devi A (2021). Oilfield geothermal resources of the Upper Assam Petroliferous Basin, NE India. Energy Geosci..

[7] Vishal V, Verma Y, Chandra D, Ashok D (2021). A systematic capacity assessment and classification of geologic CO_2_ storage systems in India. Int. J. Greenh. Gas Control.

[8] Wight NM, Bennett NS (2015). Geothermal energy from abandoned oil and gas wells using water in combination with a closed wellbore. Appl. Therm. Eng..

[9] Hu X, Banks J, Guo Y, Liu WV (2021). Retrofitting abandoned petroleum wells as doublet deep borehole heat exchangers for geothermal energy production—a numerical investigation. Renew. Energy.

[10] Nian Y-L, Cheng W-L (2018). Insights into geothermal utilization of abandoned oil and gas wells. Renew. Sustain. Energy Rev..

[11] Bu X, Ma W, Li H (2012). Geothermal energy production utilizing abandoned oil and gas wells. Renew. Energy.

[12] Bachu S (2015). Review of CO_2_ storage efficiency in deep saline aquifers. Int. J. Greenh. Gas Control.

[13] Cao C (2020). A review of CO_2_ storage in view of safety and cost-effectiveness. Energies.

[14] Wang K, Yuan B, Ji G, Wu X (2018). A comprehensive review of geothermal energy extraction and utilization in oilfields. J. Pet. Sci. Eng..

[15] Audus H (1997). Greenhouse gas mitigation technology: An overview of the CO_2_ capture and sequestration studies and further activities of the IEA Greenhouse Gas R&D Programme. Energy.

[16] Holloway S (2005). Underground sequestration of carbon dioxide—a viable greenhouse gas mitigation option. Energy.

[17] Metz, B., Davidson, O., de Coninck, H., Loos, M. & Meyer, L. IPCC Special Report on Carbon Dioxide Capture and Storage (2005).

[18] Ajayi T, Gomes JS, Bera A (2019). A review of CO2 storage in geological formations emphasizing modeling, monitoring and capacity estimation approaches. Pet. Sci..

[19] Gale J (2004). Geological storage of CO2: What do we know, where are the gaps and what more needs to be done?. Energy.

[20] Li Z, Dong M, Li S, Huang S (2006). CO2 sequestration in depleted oil and gas reservoirs—caprock characterization and storage capacity. Energy Convers. Manag..

[21] Godec M, Kuuskraa V, Van Leeuwen T, Stephen Melzer L, Wildgust N (2011). CO_2_ storage in depleted oil fields: The worldwide potential for carbon dioxide enhanced oil recovery. Energy Procedia.

[22] Yang W, Peng B, Wu M, Li J, Ni P (2016). Evaluation for CO_2_ Geo-storage Potential and Suitability in Dagang Oilfield. Energy Procedia.

[23] Cook P (2014). Geologically Storing Carbon: Learning from the Otway Project Experience.

[24] Hedley BJ, Davies RJ, Mathias SA, Hanstock D, Gluyas JG (2013). Uncertainty in static CO_2_ storage capacity estimates: Case study from the North Sea, UK: Modeling and Analysis: Uncertainty in static CO_2_ storage capacity estimates. Greenh. Gases Sci. Technol..

[25] Tuli, B., Mallya, H. & Yadav, D. Assessing India’s CO_2_ Storage Potential: A Critical Analysis of What Lies Beyond the Theoretical Potential. New Delhi: Council on Energy, Environment and Water (2023).

[26] Draft 2030 Roadmap for Carbon Capture Utilization and Storage (CCUS) for Upstream E&P Companies. (2021).

[27] Dwivedi, A. K. Petroleum Exploration in India: A perspective and Endeavours. *Proc. Indian Natl. Sci. Acad.* **82** (2016).

[28] Medlicott HB (1865). The coal of Assam, results of a brief visit to the coalfields that province in 1865; with geological note on Assam and the hills to the south of it. Mem. Geol. Surv. India.

[29] Mallet FR (1876). The coal fields of the Naga Hills bordering the Lakhimpur and Sibsagar districts, Assam: India. Mem. Geol. Surv. India.

[30] Evans, P. Tertiary succession in Assam. *Min. Geol. Inst. India* 155–260 (1932).

[31] Evans P (1964). The tectonic framework of Assam. Geol. Soc. India.

[32] Bhandari LL, Fuloria RC (1973). Stratigraphy of Assam Valley, India. AAPG Bull..

[33] Ranga Rao A (1983). Geology and hydrocarbon potential of a part of Assam-Arakan basin and its adjacent region. Pet Asia J..

[34] Bharali, B. & Gogoi, N. Sand Development Pattern Within the Paleocene - Lower Eocene Sediments Along the Shelf Areas of Upper Assam Basin - A Study Incorporating New Subsurface Information; #50556 (2012).

[35] Asante J, Ampomah W, Rose-Coss D, Cather M, Balch R (2021). Probabilistic assessment and uncertainty analysis of CO_2_ storage capacity of the morrow B sandstone—Farnsworth field unit. Energies.

[36] USDOE. Carbon Sequestration Atlas of the United States and Canada. 3rd ed., (2010).

[37] Goodman A (2011). U.S. DOE methodology for the development of geologic storage potential for carbon dioxide at the national and regional scale. Int. J. Greenh. Gas Control.

[38] Ringrose, P. Geological Storage of CO_2_ A) Processes, Capacity and Constraints.

[39] Celia MA, Bachu S, Nordbotten JM, Bandilla KW (2015). Status of CO_2_ storage in deep saline aquifers with emphasis on modeling approaches and practical simulations: Status of CO_2_ storage in deep saline aquifers. Water Resour. Res..

[40] Handique GK, Bharali B (1981). Temperature distribution and its relation to hydrocarbon accumulation in Upper Assam Valley, India: Geologic notes. AAPG Bull..

[41] Bharali, B. & Borgohain, P. Few characteristics of Tipam sandstone formation within oilfield areas of Upper Assam: A study based on wireline log data. 36–45 (2013).

[42] Borgohain P, Borah C, Gilfellon GB (2010). Sandstone diagenesis and its impact on reservoir quality of the Arenaceous Unit of Barail Group of an oilfield of Upper Assam Shelf, India. Curr. Sci..

[43] Bora, D. S., Baruah, N., Shrivastva, C. & Bharali, B. Depositional Environment and Sequence Stratigraphy of Eocene Reservoirs, Assam Shelf, India: A Multiwell Log Study. in *All Days* SPE-128668-MS (SPE, Mumbai, India, 2010). 10.2118/128668-MS.

[44] CSLF (Carbon Sequestration Leadership Forum),. Task Force for Review and Identification of Standards for CO2 Storage Capacity Estimation. (2010).

[45] NETL, Carbon Sequestration Atlas of the United States and Canada. (2009).

[46] Gorecki, C. D. *et al.* Development of Storage Coefficients for Determining the Effective CO_2_ Storage Resource in Deep Saline Formations. in *All Days* SPE-126444-MS (SPE, San Diego, California, USA, 2009). 10.2118/126444-MS.

[47] Gorecki CD, Ayash SC, Liu G, Braunberger JR, Dotzenrod NW (2015). A comparison of volumetric and dynamic CO_2_ storage resource and efficiency in deep saline formations. Int. J. Greenh. Gas Control.

[48] Ye J (2023). Evaluation of geological CO_2_ storage potential in Saudi Arabian sedimentary basins. Earth-Sci. Rev..

[49] Tixier MP (1949). Evaluation of permeability from electric log resistivity gradients. Oil Gas J..

[50] Timur A (1968). An investigation of permeability, porosity and residual water saturation relationships for sandstone reservoirs. Log Anal..

[51] Coats GR, Dumanoir JL (1974). A new approach to improved log-derived permeability. Log Anal..

[52] Singh NP (2019). Permeability prediction from wireline logging and core data: A case study from Assam-Arakan basin. J. Pet. Explor. Prod. Technol..

[53] Gupta ML (1982). Heat flow in the Indian Peninsula—its geological and geophysical implications. Tectonophysics.

[54] Razdan PN, Agarwal RK, Singh R (2008). Geothermal energy resources and its potential in India. J. Earth Sci. India.

[55] Agemar T (2022). Bottom hole temperature correction based on empirical correlation. Geothermics.

[56] Santoyo E, Garcia A, Espinosa G, Hernandez I, Santoyo S (2000). STATIC_TEMP: A useful computer code for calculating static formation temperatures in geothermal wells. Comput. Geosci..

[57] Schölderle F, Götzl G, Einsiedl F, Zosseder K (2022). Uncertainty assessment of corrected bottom-hole temperatures based on Monte Carlo techniques. Energies.

[58] Zare-Reisabadi M, Kamali MR, Mohammadnia M, Shabani F (2015). Estimation of true formation temperature from well logs for basin modeling in Persian Gulf. J. Pet. Sci. Eng..

[59] Harrison, W. E., Luza, K. V., Prater, M. L. & Chueng, P. K. Geothermal resource assessment of Oklahoma. *Okla. Geol. Surv. Spec. Publ* (1983).

[60] Waples DW, Pedersen MR (2004). Evaluation of Horner plot-corrected log-derived temperatures in the Danish Central Graben, North Sea. Nat. Resour. Res..

[61] Hackstein FV, Madlener R (2021). Sustainable operation of geothermal power plants: Why economics matters. Geotherm. Energy.

[62] Gringarten Alain C. Reservoir lifetime and heat recovery factor in geothermal aquifers used for urban heating. (1978).

[63] Gringarten AC, Sauty JP (1975). A theoretical study of heat extraction from aquifers with uniform regional flow. J. Geophys. Res..

[64] Deb, S. S. & Barua, I. Depositional Environment, Reservoir Characteristics and Extent of Sediments of Langpar & Lakadong+Therria in Chabua Area of Upper Assam Basin. 8th Biennial International Conference&Exposition on Petroleum Geophysics, 177 (2010).

[65] Thorsteinsson, H., Augustine, C., Anderson, B. J., Moore, M. C. & W. Testera, J. The impacts of drilling and reservoir technology advances on egs exploitation. *Proc. Thirty-Third Workshop Geotherm. Reserv. Eng. Stanf. Univ. Stanf. Calif.* **SGP-TR-185**, (2008).

[66] Risktec Solutions Limited. *Bowtie Analysis - Carbon Storage SECURe Project*. (2021).

[67] Tucker O, Holley M, Metcalfe R, Hurst S (2013). Containment risk management for CO_2_ storage in a depleted gas field, UK North Sea. Energy Procedia.

[68] Askarova A (2023). An overview of geological CO_2_ sequestration in oil and gas reservoirs. Energies.

[69] Zapata Y (2020). CO2 geological storage: Critical insights on plume dynamics and storage efficiency during long-term injection and post-injection periods. J. Nat. Gas Sci. Eng..

[70] Deng H, Stauffer PH, Dai Z, Jiao Z, Surdam RC (2012). Simulation of industrial-scale CO_2_ storage: Multi-scale heterogeneity and its impacts on storage capacity, injectivity and leakage. Int. J. Greenh. Gas Control.

[71] Gruson J-F (2015). Techno-economic assessment of four CO_2_ storage sites. Oil Gas Sci. Technol. Rev. D’IFP Energy Nouv..

[72] Guzović Z, Lončar D, Ferdelji N (2010). Possibilities of electricity generation in the Republic of Croatia by means of geothermal energy. Energy.

[73] Chamorro CR (2012). World geothermal power production status: Energy, environmental and economic study of high enthalpy technologies. Energy.

[74] Sanyal, S. K. Cost of geothermal power and factors that affect it. *Proc. Twenty-Ninth Workshop Geotherm. Reserv. Eng. Stanf. Univ. Stanf. Calif.* (2004).

