# Supplementary material for: Integrated coupled assessment of geostorage and geothermal prospects in the oil fields of Upper Assam Basin
Source: Sci Rep. 2024 May 29;14:12390. doi: 10.1038/s41598-024-60292-3 (PMC11137029; doi:10.1038/s41598-024-60292-3)
Supplement: Supplementary file 1 — Supplementary Information. [file 41598_2024_60292_MOESM1_ESM.docx]

| **Formation** | **Sand Ranges** | **Resistivity (Ohm-m)** | **Phie (%)** | **S_w_ (%)** |
| --- | --- | --- | --- | --- |
| **Nurpuh** | 3550 (Top) | 15 to 22 | 08 to 12 | 60 |
|  | 3576 (Bottom) |  |  |  |
| **Lakadong+Therria** | 3610 (Top) | 400 Ohm-m to 3612 (m) | 15 to 22 | 20 upto 3612 |
|  | 3621 (Bottom) | 30-60 Ohm-m below |  | 30 to 40 below |
|  | 3624 (Top) | 30-35 | 10 to 12 | 40 |
|  | 3626.5 (Bottom) |  |  |  |
|  | 3629 (Top) | 200-1000 | 20 to 24 | 5 to 20 |
|  | 3649 (Bottom) |  |  |  |
|  | 3684 (Top) | >2000 | 15 to 18 | 5 to 10 |
|  | 3699 (Bottom) |  |  |  |
|  | 3706 (Top) | 300 | 12 to 14 | 15 to 20 |
|  | 3708 (Bottom) |  |  |  |
|  | 3713(Top) | 30 to 35 | 08 to 10 | 40 |
|  | 3715(Bottom) |  |  |  |
|  | 3717.5(Top) | 30 to 90 (upto 3721.5) | 8 to 12 (upto 3721.5) | 30 to 40 |
|  | 3726.5(Bottom) | 40 | 15 |  |
|  | 3735.5(Top) | 1500 (upto 3741) | 16 (upto 3741) | 10 to 20 |
|  | 3744.5(Bottom) | 60-180 | 8 |  |
|  | 3747(Top) | 100 to 500 (upto 3757) | 14-16 | 20 (upto3757) |
|  | 3772.5(Bottom) | 25-50 |  | 50-70 |

**Well-Log Data of Seventeen WELLS in this Study**

1. **WELL F**
2. **WELL C**

| **Formation** | **Sand Ranges** | **Resistivity (Ohm-m)** | **Phie (%)** | **S_w_ (%)** |
| --- | --- | --- | --- | --- |
| **Nurpuh** | 3618(Top) | 20-30 | 12 | 40 |
|  | 3623(Bottom) |  |  |  |
|  | 3642.5(Top) | 15-20 | 15 | 50-60 |
|  | 3652(Bottom) |  |  |  |
|  | 3653.5 | 15-20 | 12 | 45-55 |
|  | 3666 |  |  |  |
| **Lakadong+Therria** | 3696(Top) | 30-35 | 20 | 40 |
|  | 3701.5(Bottom) |  |  |  |
|  | 3709.7(Top) | 150 | 24 | 20-25 |
|  | 3712(Bottom) |  |  |  |
|  | 3714.5(Top) | 50-150 | 18-22 | 20-35 |
|  | 3717.2(Bottom) |  |  |  |
|  | 3719.5(Top) | 100-200 | 18-22 | 15-25 |
|  | 3728(Bottom) |  |  |  |
|  | 3729(Top) | 300-1000 | 25 | 10 to 15 |
|  | 3737.8(Bottom) |  |  |  |
|  | 3780(Top) | 1000 to >2000 | 22-25 | 5 |
|  | 3795.5(Bottom) |  |  |  |
|  | 3812(Top) | 40-60 | 12 | 40-50 |
|  | 3814.5(Bottom) |  |  |  |

1. **WELL D**

| **Formation** | **Sand Ranges** | **Resistivity (Ohm-m)** | **Phie (%)** | **S_w_ (%)** |
| --- | --- | --- | --- | --- |
| **Lakadong+Therria** | 4054(Top) | 25-40 |  |  |
|  | 4070(Bottom) |  |  |  |
|  | 4149(Top) | 10 to 18 |  |  |
|  | 4158(bottom) |  |  |  |
|  | 4828(Top) | 200 | <5 | 70-90 |
|  | 4838(Bottom) |  |  |  |
|  | 4839(Top) | 30-100 | 7 to 10 | 60-70 |
|  | 4842(Bottom) |  |  |  |
|  | 4842.5(Top) |  |  |  |
|  | 4845.5(Bottom) |  |  |  |
|  | 4848.5(Top) | 200-1000 | 5 to 10 | 30 (upto 4852.5m) |
|  | 4854(Bottom) |  |  | 60 |
|  | 4855 | 35-120 |  |  |
|  | 4858 |  |  |  |
|  | 4864 | 12 to 20 | 12 | 100 |
|  | 4869 |  |  |  |
|  | 4874 | 100-150 | 5 to 8 | 90-100 |
|  | 4878 |  |  |  |
|  | 4882.5(Top) | 25-40 | 5 to 8 |  |
|  | 4884.5(Bottom) |  |  |  |
|  | 4889.5(Top) |  |  |  |
|  | 4890.5(Bottom) |  |  |  |
|  | 4896 (Top) | 60 | 5 | 75 |
|  | 4897.5 (Bottom) |  |  |  |
|  | 4902( Top) |  |  |  |

1. **WELL K**

| **Formation** | **Sand Ranges (m)** | **Resistivity (Ohm-m)** | **Phie (%)** | **S_w_ (%)** |
| --- | --- | --- | --- | --- |
| **Nurpuh** | 3737.5(Top) | 10 to 17 | 5 to 15 | 70 to 80 |
|  | 3741.5(Bottom) |  |  |  |
|  | 3754(Top) | 10 to 20 | 10 to 15 | 55 to 80 |
|  | 3771(Bottom) |  |  |  |
| **Lakadong+Therria** | 3823 (Top) | 15 to 20 | 15 to 25 | 40-70 |
|  | 3829 (Bottom) |  |  |  |
|  | 3831 (Top) | 20 to 300 | 12 to 25 | 20 to 60 |
|  | 3838.5 (Bottom) |  |  |  |
|  | 3886 (Top) | 600 to 2000 | 23 to 25 | 5 to 10 |
|  | 3897.5 (Bottom) |  |  |  |
|  | 3904.3 (Top) | 10 to 20 | 3 to 8 | 50 to 55 |
|  | 3909 (Bottom) |  |  |  |
|  | 3913.5 (Top) | 15 to 45 | 10 to 20 | 57-70 |
|  | 3922 (Bottom) |  |  |  |

1. **WELL O**

| **Formation** | **Sand Ranges (m)** | **Resistivity (Ohm-m)** | **Phie (%)** | **S_w_ (%)** |
| --- | --- | --- | --- | --- |
| **Barail** | 3077 (Top) | 7 to 20 | 8 | 80 |
|  | 3083(Bottom) |  |  |  |
|  | 3099(Top) | 40 | 8 | 30 |
|  | 3101(Bottom) |  |  |  |
|  | 3117.5(Top) | 50 | 12 | 35 |
|  | 3120(Bottom) |  |  |  |
|  | 3133.5(Top) | 6 to 10 | 8 | 60 |
|  | 3152(Bottom) |  |  |  |
|  | 3167(Top) | 15 | 8 | 60 |
|  | 3171(Bottom) |  |  |  |
|  | 3188(Top) | 10 | 6 | 80 |
|  | 3203(Bottom) |  |  |  |
| **Lakadong+Therria** | 4418(Top) | 20 to 300 | 5 to 9 | 60 to 70 |
|  | 4428(Bottom) |  |  |  |
|  | 4429(Top) | 20 to 200 | 8 to 12 | 60 to 80 |
|  | 4433.2(Bottom) |  |  |  |
|  | 4434.5 (Top) | 40 to 50 | 8 to 12 | 40 to 60 |
|  | 4436.5 (Bottom) |  |  |  |
|  | 4439(Top) | 1000 | 8 to 10 | 40 |
|  | 4441.8(Bottom) |  |  |  |
|  | 4442.5 (Top) | 30 to 60 | 8 to 12 | 50 to 60 |
|  | 4444(Bottom) |  |  |  |
|  | 4444.8 (Top) |  |  |  |
|  | 4445.8 (Bottom) |  |  |  |
|  | 4479 (Top) | 28 to 50 | 9 | 90 to 100 |
|  | 4485(Bottom) |  |  |  |
|  | 4512.5 (Top) | 30 to 50 | 8 | 95 |
|  | 4519.5 (Bottom) |  |  |  |
|  | 4525.5(Top) | 35 to 70 | 3 to 5 | 100 |
|  | 4530(Bottom) |  |  |  |

1. **WELL J**

| **Formation** | **Sand Ranges (m)** | **Resistivity (Ohm-m)** | **Phie (%)** | **S_w_ (%)** |
| --- | --- | --- | --- | --- |
| **Lakadong+Therria** |  |  |  |  |
|  | 3554.5 (Top) | 15 to 40 | 5 to 10 | 50 to 70 |
|  | 3558 (Bottom) |  |  |  |
|  | 3578 (Top) | 15 to 25 | 8 | 70 to 75 |
|  | 3581.2 (Bottom) |  |  |  |
|  | 3584.5 (Top) | 18 to 20 | 5 to 15 | 70 |
|  | 3587.8 (Bottom) |  |  |  |
|  | 3592(Top) | 30 to 100 | 10 to 18 | 30 to 40 |
|  | 3596 (Bottom) |  |  |  |
|  | 3597.8 (Top) | 55 | 14 | 50 |
|  | 3599.3 (Bottom) |  |  |  |
|  | 3620.5 (Top) | 10 to 20 | 15 to 20 | 50 to 90 |
|  | 3623 (Bottom) |  |  |  |
|  | 3635.5 (Top) | 12 | 15 to 22 | 80 to 90 |
|  | 3638.2 (Bottom) |  |  |  |
|  | 3649 (Top) | 70 | 10 | 75 to 80 |
|  | 3650.2 (Bottom) |  |  |  |
|  | 3652.5 (Top) | 20 to 30 | 10 to 12 | 100 |
|  | 3670 (Bottom) |  |  |  |

1. **WELL I**

| **Formation** | **Sand Ranges (m)** | **Resistivity (Ohm-m)** | **Phie (%)** | **S_w_ (%)** |
| --- | --- | --- | --- | --- |
| **Kopili** | 3184 (Top) | 8 to 18 | 12 to 25 | 55 to 80 |
|  | 3192 (Bottom) |  |  |  |
| **Nurpuh** | 3506 (Top) | 15 to 22 | 16 to 18 | 60 to 75 |
|  | 3510 (Bottom) |  |  |  |
|  | 3517.5 (Top) | 18 to 30 | 14 to 18 | 60 to 70 |
|  | 3523.3 (Bottom) |  |  |  |
|  | 3536.3 (Top) | 50 to 100 | 18 to 22 | 35 to 65 |
|  | 3540 (Bottom) |  |  |  |
| **Lakadong + Therria** | 3563.4 (Top) | 40 to 90 | 25 (upto 3568) | 30 to 50 |
|  | 3569.8 (Bottom) |  | 14 |  |
|  | 3573.4 (Top) | 40 | 18 to 20 | 40 to 60 |
|  | 3574.8 (Bottom) |  |  |  |
|  | 3579.5 (Top) | 30 to 300 | 15 to 25 | 20 to 50 |
|  | 3582.5 (Bottom) |  |  |  |
|  | 3614.6 (Top) | 30 to 90 | 20 to22 | 35 to 50 |
|  | 3617.2 (Bottom) |  |  |  |
|  | 3622 (Top) | 300 | 6 | 40 |
|  | 3623.2 (Bottom) |  |  |  |
|  | 3626.5 (Top) | 12 to 18 | 18 to 22 | 60 to 100 |
|  | 3634 (Bottom) |  |  |  |
|  | 3639.5 (Top) | 8 to 25 | 14 to 20 | 80 to 100 |
|  | 3659(Bottom) |  |  |  |

1. **WELL M2**

| **Formation** | **Sand Ranges (m)** | **Resistivity (Ohm-m)** | **Phie (%)** | **S_w_ (%)** |
| --- | --- | --- | --- | --- |
| **Lakadong+Therria** | 4185.6 (Top) | 20 to 250 | 5 to 10 | 100 |
|  | 4198.5 (Bottom) |  |  |  |
|  | 4204 (Top) | 25 | 8 to 12 | 80 to 92 |
|  | 4206 (Bottom) |  |  |  |
|  | 4210 (Top) | 15 | 15 | 100 |
|  | 4215 (Bottom) |  |  |  |
|  | 4217.5 (Top) | 12 to 18 | 10 to 18 | 100 |
|  | 4227.5 (Bottom) |  |  |  |

1. **WELL P**

| **Formation** | **Sand Ranges (m)** | **Resistivity (Ohm-m)** | **Phie (%)** | **S_w_ (%)** |
| --- | --- | --- | --- | --- |
| **Barail** | 3350 (Top) | 10 to 15 (Upto 3357.5) | 5 to 12 (upto 3362) | 60 to 75 (upto 3357.5) |
|  |  | 15 to 40 (Upto 3387) | 20 to 22 (upto 3391 | 30 to 60 (upto 3387.5) |
|  | 3400 (Bottom) | 10 to 12 (3400) | 10 to 13(upto 3400) | 70 to 80 (upto 3400) |
|  | 3412 (Top) | 5 to 8 | 14 to 25 | 80 to 100 |
|  | 3512(Bottom) |  |  |  |
| **Lakadong+Therria** | 4326(Top) | 15 to 100 | 10 to 12 | 70 to 100 |
|  | 4328.4(Bottom) |  |  |  |
|  | 4331.5 (Top) | 15 to 100 | 13 | 100 |
|  | 4333.5 (Bottom) |  |  |  |
|  | 4349.5 (Top) | 10 to 30 | 13 to 18 | 80 to 95 |
|  | 4358.4 (Bottom) |  |  |  |
|  | 4362.5 (Top) | 20 | 12 | 80 to 95 |
|  | 4364.2(Bottom) |  |  |  |
|  | 4372.8 (Top) | 15 to 20 | 12 to 20 | 50 to 100 |
|  | 4382.5(Bottom) |  |  |  |
|  | 4389.5 (Top) | 10 to 30 | 16 to 20 | 80 to 100 |
|  | 4423 (Bottom) |  |  |  |

| **Formation** | **Sand Ranges (m)** | **Resistivity (Ohm-m)** | **Phie (%)** | **S_w_ (%)** |
| --- | --- | --- | --- | --- |
| **Lakadong + Therria** | 3589 (Top) | 15 to 30 | 4 to 10 | 55 to 75 |
|  | 3597.5 (Bottom) |  |  |  |
|  | 3601.3 (Top) | 35 | 10 | 40 to 50 |
|  | 3602.5 (Bottom) |  |  |  |
|  | 3606 (Top) | 200 to 1000 | 18 to 28 | 10 to 20 |
|  | 3609.5 (Bottom) |  |  |  |
|  | 3631 (Top) | 30 to 45 | 18 | 60 |
|  | 3633 (Bottom) |  |  |  |
|  | 3640.5 (Top) | 30 | 12 | 70 |
|  | 3641.8 (Bottom) |  |  |  |
|  | 3645.8 (Top) | 15 to 30 | 13 | 65 to 80 |
|  | 3648 (Bottom) |  |  |  |
|  | 3649.9 (Top) | 25 to 30 | 8 to 12 | 70 |
|  | 3652 (Bottom) |  |  |  |
|  | 3662.2 (Top) | 80 to 100 | 12 | 40 to 60 |
|  | 3665.3 (Bottom) |  |  |  |

1. **WELL E**

1. **Well A**

| **Formation** | **Sand Ranges (m)** | **Resistivity (Ohm-m)** | **Phie (%)** | **S_w_ (%)** |
| --- | --- | --- | --- | --- |
| **Lakadong+Therria** | 3954.7 (Top) | 8 to 18 | 10 to 13 | 70 to 80 |
|  | 3960 (Bottom) |  |  |  |
|  | 3981.4 (Top) | 10 to 20 | 10 to 13 | 70 to 90 |
|  | 3990.5 (Bottom) |  |  |  |
|  | 3997.5 (Top) | 20 to 60 | 18 to 25 | 30 to 40 |
|  | 4001 (Bottom) |  |  |  |
|  | 4008.5 (Top) | 8 to 10 | 20 to 25 | 75 to 90 |
|  | 4013.5 (Bottom) |  |  |  |
|  | 4025.3 (Top) | 7 to 12 | 20 to 22 | 80 to 100 |
|  | 4028 (Bottom) |  |  |  |
|  | 4031.5 (Top) | 20 to 25 | 16 to 71 | 100 |
|  | 4032.8 (Bottom) |  |  |  |
|  | 4046 (Top) | 20 to 100 | 12 to 16 | 50 to 90 |
|  | 4053 (Bottom) |  |  |  |
| **Langpar** | 4073.7 (Top) | 15 to 20 | 15 | 60 to 100 |
|  | 4077 (Bottom) |  |  |  |

1. **WELL L**

| **Formation** | **Sand Ranges (m)** | **Resistivity (Ohm-m)** | **Phie (%)** | **S_w_ (%)** |
| --- | --- | --- | --- | --- |
| **Lakadong+Therria** | 3618.5 (Top) | 35 to 45 | 16 | 50 to 55 |
|  | 3621.5 (Bottom) |  |  |  |
|  | 3623.5 (Top) | Upto 80 | 12 to 20 | 40 |
|  | 3627.5 (Bottom) |  |  |  |
|  | 3638 (Top) | 35 to 45 | 35 to 45 | 90 |
|  | 3639.5 (Bottom) |  |  |  |
|  | 3646.5 (Top) | upto 80 | 8 to 10 | 45 |
|  | 3651.5 (Bottom) |  |  |  |
|  | 3659.5 (Top) | 18 to 25 | 18 to 25 | 80 |
|  | 3662 (Bottom) |  |  |  |
|  | 3667.5 (Top) | upto 50 | 10 to 12 | 90 |
|  | 3671.5 (Bottom) |  |  |  |
| **Langpar** | 3672 (Top) | 50 to 80 | 10 to 12 | 75 to 90 |
|  | 3674 (Bottom) |  |  |  |
|  | 3679 (Top) | upto 190 | Upto 28 | 190 |
|  | 3680 (Bottom) |  |  |  |
|  | 3687 (Top) | 60 to 120 | 4 to 8 | 75 to 85 |
|  | 3690.5 (Bottom) |  |  |  |
|  | 3703 (Top) | 25 to 35 | 25 to 35 | 80 to 90 |
|  | 3709 (Bottom) |  |  |  |

1. **WELL N**

| **Formation** | **Sand Ranges (m)** | **Resistivity (Ohm-m)** | **Phie (%)** | **S_w_ (%)** |
| --- | --- | --- | --- | --- |
| **Nurpuh** | 3976 | 18 to 20 | 7 to 10 | 40 |
|  | 3980 |  |  |  |
| **Lakadong+Therria** | 4009 (Top) | 30 | 17 to 20 | 40 |
|  | 4010 (Bottom) |  |  |  |
|  | 4049 (Top) | 20 to 25 | 17 to 20 | 50 to 60 |
|  | 4055 (Bottom) |  |  |  |
|  | 4059.8 (Top) | 100 | 10 to 12 | 50 |
|  | 4066 (Bottom) |  |  |  |
|  | 4069 (Top) | 25 to 32 | 8 to 10 | 50 to 65 |
|  | 4071.6 (Bottom) |  |  |  |
|  | 4080.5 (Top) | 100 | 5 | 50 |
|  | 4081.5 (Bottom) |  |  |  |
|  | 4085.1 (Top) | 40 | 5 | 60 |
|  | 4086.2 (Bottom) |  |  |  |
|  | 4091.2 (Top) | 22 to 40 | 2 to 8 | 50 to 60 |
|  | 4093.5 (Bottom) |  |  |  |
|  | 4104.5 (Top) | 30 to 80 | 4 to 12 | 50 to 70 |
|  | 4107.4 (Bottom) |  |  |  |
|  | 4110 (Top) | 30 | 12 | 45 |
|  | 4117 (Bottom) |  |  |  |
|  | 4128 (Top) | 50 | 12 to 18 | 50 |
|  | 4131 (Bottom) |  |  |  |
|  | 4133 (Top) | 15 to 60 | 12 to 15 | 52 |
|  | 4134.8 (Bottom) |  |  |  |
|  | 4151 (Top) | 15 to 18 | upto 10 | 100 |
|  | 4153.3 (Bottom) |  |  |  |
|  | 4172 (Top) | 10 to 20 | upto 15 | 100 |
|  | 4173 (Bottom) |  |  |  |
|  | 4177.2 (Top) | 6 to 15 | 18 to 22 | 100 |
|  | 4184 (Bottom) |  |  |  |
|  | 4185 (Top) | 6 to 15 | 20 to 25 | 100 |
|  | 4195 (Bottom) |  |  |  |
|  | 4197.5 (Top) | 6 to 7 | 18 to 22 | 100 |
|  | 4207 (Bottom) |  |  |  |

1. **WELL H**

| **Formation** | **Sand Ranges (m)** | **Resistivity (Ohm-m)** | **Phie (%)** | **S_w_ (%)** |
| --- | --- | --- | --- | --- |
| **Lakadong+Therria** | 3564.5 (Top) | 20 to 70 | 5 to 12 | 65 |
|  | 3572.5 (Bottom) |  |  |  |
|  | 3573 (Top) | 20 to 70 | 8 | 10 to 15 |
|  | 3579 (Bottom) |  |  |  |
|  | 3590 (Top) | 30 to 40 | 10 to 18 | 45 to 60 |
|  | 3593.3 (Bottom) |  |  |  |
|  | 3598.5 (Top) | 18 to 22 | 16 | 70 to 80 |
|  | 3600 (Bottom) |  |  |  |
|  | 3602 (Top) | 10 to 55 | 23 | 45 to 100 |
|  | 3605 (Bottom) |  |  |  |
|  | 3613 (Top) | 10 | 20 | 80 to 90 |
|  | 3615 (Bottom) |  |  |  |
|  | 3616.5 (Top) | 8 | 20 | 80 to 90 |
|  | 3620 (Bottom) |  |  |  |
|  | 3625.5 (Top) | 8 to 12 | 20 | 100 |
|  | 3627.5 (Bottom) |  |  |  |
|  | 3630.5 (Top) | 8 to 12 | 22 | 100 |
|  | 3634.5 (Bottom) |  |  |  |
|  | 3639 (Top) | 10 to 12 | 18 to 22 | 90 to 100 |
|  | 3642.5 (Bottom) |  |  |  |
|  | 3646 (Top) | 10 to 12 | 18 to 22 | 90 to 100 |
|  | 3651 (Bottom) |  |  |  |

1. **WELL G**

| **Formation** | **Sand Ranges (m)** | **Resistivity (Ohm-m)** | **Phie (%)** | **S_w_ (%)** |
| --- | --- | --- | --- | --- |
| **Lakadong+Therria** | 4431 (Top) | 20 to 30 | 8 to 10 | 60 to 70 |
|  | 4433 (Bottom) |  |  |  |
|  | 4434 (Top) | 25 to 200 | 5 to 10 | 90 to 100 |
|  | 4439 (Bottom) |  |  |  |
|  | 4454.5 (Top) | 150 to 300 | 10 to 15 | 70 to 80 |
|  | 4459.5 (Bottom) |  |  |  |
|  | 4463.5 (Top) | 40 to 60 | 5 to 10 | 60 to 90 |
|  | 4465.5 (Bottom) |  |  |  |
|  | 4495.5 (Top) | 70 to 150 | 10 to 18 | 30 to 40 |
|  | 4500.5 (Bottom) |  |  |  |

1. **WELL B**

| **Formation** | **Sand Ranges (m)** | **Resistivity (Ohm-m)** | **Phie (%)** | **S_w_ (%)** |
| --- | --- | --- | --- | --- |
| **Lakadong+Therria** | 3473.5 (Top) | 60 | 9 to 12 | 30 |
|  | 3476 (Bottom) |  |  |  |
|  | 3481.6 (Top) | 25 | 9 to 15 | 20 |
|  | 3483.3 (Bottom) |  |  |  |
|  | 3490 (Top) | 25 | 15 | 40 |
|  | 3491 (Bottom) |  |  |  |
|  | 3493 (Top) | 40 | 13 | 60 to 70 |
|  | 3494 (Bottom) |  |  |  |
|  | 3500 (Top) | 200 | 15 to 17 | 25 |
|  | 3502.3 (Bottom) |  |  |  |
|  | 3504.6 (Top) | 100 | 10 to 12 | 30 to 40 |
|  | 3506.3 (Bottom) |  |  |  |
|  | 3512 (Top) | 800 to 900 | 22 | 10 |
|  | 3521 (Bottom) |  |  |  |
|  | 3523.5 (Top) | 45 | 12 | 70 |
|  | 3525 (Bottom) |  |  |  |
|  | 3526 (Top) | 30 | 8 | 80 |
|  | 3527.8 (Bottom) |  |  |  |
|  | 3535.5 (Top) | 20 | 12 | 100 |
|  | 3539.5 (Bottom) |  |  |  |
| **Langpar** | 3566 (Top) | 90 | 15 to 20 | 30 to 50 |
|  | 3567 (Bottom) |  |  |  |

1. **WELL M1**

| **Formation** | **Sand Ranges (m)** | **Resistivity (Ohm-m)** | **Phie (%)** | **S_w_ (%)** |
| --- | --- | --- | --- | --- |
| **Lakadong+Therria** | 4533.7 (Top) | 100 to 150 | 4 to 18 | 35 to 75 |
|  | 4537 (Bottom) |  |  |  |
|  | 4538.5 (Top) | 300 to 2000 | 4 to 6 | 25 to 55 |
|  | 4541.5 (Bottom) |  |  |  |
|  | 4543 (Top) | 100 to 1000 | 10 to 14 | 15 to 30 |
|  | 4551 (Bottom) |  |  |  |
|  | 4555.2 (Top) | 50 to 500 | 5 to 14 | 35 to 60 |
|  | 4562.5 (Bottom) |  |  |  |
|  | 4569 (Top) | 90 | 8 to 12 | 45 to 55 |
|  | 4572 (Bottom) |  |  |  |
|  | 4574 (Top) | 20 to 90 | 8 to 12 | 45 to 55 |
|  | 4576 (Bottom) |  |  |  |
|  | 4589.5 (Top) | 15 to 50 | 5 to 6 | 80 to 100 |
|  | 4595.5 (Bottom) |  |  |  |
| **Langpar** | 4649 (Top) | 10 to 50 | 6 to 8 | 100 |
|  | 4674.5 (Bottom) |  |  |  |

Abbreviations:

S_w_ :Water Saturation

Phie(%): Porosity
